# *CWGCNA*: an R package to perform causal inference from the *WGCNA* framework

**DOI:** 10.1093/nargab/lqae042

**Published:** 2024-04-25

**Authors:** Yu Liu

**Affiliations:** Laboratory of Pathology, Center for Cancer Research, National Cancer Institute, Bethesda, MD 20892, USA

## Abstract

*WGCNA* (weighted gene co-expression network analysis) is a very useful tool for identifying co-expressed gene modules and detecting their correlations to phenotypic traits. Here, we explored more possibilities about it and developed the R package *CWGCNA* (causal *WGCNA*), which works from the traditional *WGCNA* pipeline but mines more information. It couples a mediation model with *WGCNA*, so the causal relationships among *WGCNA* modules, module features, and phenotypes can be found, demonstrating whether the module change causes the phenotype change or *vice versa*. After that, when annotating the module gene set functions, it uses a novel network-based method, considering the modules' topological structures and capturing their influence on the gene set functions. In addition to conducting these biological explorations, *CWGCNA* also contains a machine learning section to perform clustering and classification on multi-omics data, given the increasing popularity of this data type. Some basic functions, such as differential feature identification, are also available in our package. Its effectiveness is proved by the performance on three single or multi-omics datasets, showing better performance than existing methods. *CWGCNA* is available at: https://github.com/yuabrahamliu/CWGCNA.

## Introduction

Many methods have been developed for high-throughput omics data analysis. Among them, *WGCNA* (weighted gene co-expression network analysis) is one of the most popular tools. It clusters the genes into highly co-expressed groups (modules) with a soft thresholding strategy, avoiding information loss and enhancing result robustness (1). Then, the modules are linked to biological meanings via their correlation to the phenotypic traits.

This framework presents a clear relationship between the phenotype and its underlying genes, proven to be very helpful in many areas (2–4). Here, we explored more possibilities about it and developed the R package *CWGCNA* (causal *WGCNA*), which works from the traditional *WGCNA* pipeline but mines more information.

It conducts a mediation analysis among the phenotype, the *WGCNA* module, and the module features so that their undirected correlations revealed by *WGCNA* can be clarified as directed ones, i.e. their causal relationships can be found, demonstrating whether the module change causes the phenotype change or *vice versa*.

The core of *CWGCNA* is mediation analysis. It is a method for understanding mechanisms and identifying intervention points (5–7). Concretely, it assesses the extent to which the effect of one variable (exposure) on another (outcome) is mediated by some intermediate variable (mediator). As such, notions of mediation concern causality (5). Hence, social scientists have used it to reveal causal mechanisms and design policy interventions (7,8). Besides, it has applications in environmental and epidemiological studies, such as the observation that 8-isoprostane, an oxidative stress biomarker, mediates the effect of plasticizer exposure on preterm birth (9).

However, this method is not used in high-throughput omics data, which is surprising because finding the cause and mediators of a disease and applying intervention is the core of biomedical studies, and mediation analysis provides a unique approach. To fill this gap, we combined this method with *WGCNA*, helping biologists to easily incorporate it into their omics analysis.

In detail, *CWGCNA* uses the *WGCNA* module, the module features, and the phenotype to construct mediation models. For RNA expression data, a module means a group of genes whose transcription profiles are highly correlated, and the features are the genes in that group (1). The mediation models use the module's eigengene (the PC1 value, i.e. the first principal component of all the genes of the module), the gene expression level, and the phenotypic traits to test two causal directions: (i) the forward direction of "module→module feature→phenotype", which means the module drives the phenotype via its feature, and (ii) the reverse direction of "phenotype→module feature→module", which means the module changes following the phenotype. By these tests, the cause and mediators of a phenotype can be clarified.

In addition, *CWGCNA* contains other functions for omics analysis, such as network-based gene set function annotation, multiple CCA (canonical component analysis)-based multi-omics data clustering, and ensemble-based multi-omics classification, which also have unique advantages.

To test *CWGCNA*, we applied it to three datasets. Results show that it performs better than existing methods. It can enhance the power of *WGCNA* and provide more possibilities for exploring omics data.

## Materials and methods

### Data collection and preprocessing (*imputemeta* and *probestogenes*)

The Infinium 27K and 450K data on control and preeclampsia human placentas were obtained from 10 GEO datasets: GSE31781 (10), GSE36829, GSE59274 (11), GSE74738 (12), GSE69502 (13), GSE98224 (14,15), GSE125605, GSE100197, GSE75196 and GSE73375. Then, we used *SeSAMe* to perform data preprocessing and merged the datasets so that only the overlapping probes shared by the Illumina 27K and 450K datasets were kept (16,17). The batch difference was adjusted via *ComBat* with GSE98224 data as the reference (18).

The above 10 datasets contained 359 placenta samples (258 control and 101 preeclampsia) and provided the gestational weeks for all of them (from 8 weeks to 44.6 weeks). In addition, 210 of the 359 samples had baby gender information, and 102 of the 359 samples had ethnicity information. To impute the missing values for baby gender and ethnicity, we used the gender prediction and ethnicity prediction models provided by *SeSAMe*, and the function *imputemeta* in our package. After that, the dataset covered the 359 samples' complete phenotypic data on four variables: preeclampsia/control group, gestational week, baby gender, and ethnicity. More descriptions about the missing value imputation with *SeSAMe* and *imputemeta* were provided in Supplementary Data.

The data for the 419 LUAD (lung adenocarcinoma) and the 752 BRCA (breast invasive carcinoma) cancer samples were from TCGA, with their RNA-seq read counts, 450K DNA methylation (DNAm) beta values, miRNA-seq read counts, and clinical data downloaded. The BRCA samples' BRCA PAM50 subtype information was from the R package *TCGAbiolinks* (19). The preprocessing of these data involved the functions *imputemeta* and *probestogenes* in our package, and the details were described in Supplementary Data.

### ANOVA analysis (*featuresampling*)

The function *featuresampling* in our package was used to perform type-Ⅲ ANOVA to determine the confounding factors. It constructed a linear regression model to predict each candidate feature with the phenotypic variables. Then, it used type-Ⅲ ANOVA on the model to calculate the MSS (mean sum of the square), *F* statistic, and *P*-value for each phenotypic variable. Finally, each variable got the MSSs, *F* statistics and *P*-values across all the models constructed for all the features, and their means were used to represent the value for the whole dataset.

### Causal *WGCNA* analysis (*diffwgcna*)

The function *diffwgcna* performed causal *WGCNA* analysis. First, it used the traditional *WGCNA* method to calculate *WGCNA* modules from the data. Then, it compared the module eigengenes between sample groups by *limma*, identifying the modules related to the group difference. Furthermore, within each module, *limma* was also used on its nodes (features) to find the features related to the sample group difference.

This *limma* regression connected the modules and features with the sample group difference. However, this connection was undirected, ignoring the potential causal relationships among the sample groups, module features, and modules. Hence, *diffwgcna* used mediation models to find any directed connections, which could be triggered by setting its parameter *mediation* as TRUE. The mediation models were the core of causal *WGCNA* and were constructed with the product method coupled with IPW (inverse probability weighting) (6,9,20–22).

The mediation models were constructed after *diffwgcna* identified the inter-group differential features within each module. For RNA expression data, a module meant a group of genes whose transcription profiles were highly correlated, and the features were the genes in that group (1). A module's eigengene and a gene's expression level were used when constructing a mediation model, and for each feature in a specific module, *diffwgcna* built two models. The first one tested the mediation relationship of "module→module feature→group", which meant the module caused the sample group difference using its feature as the mediator. The second tested the opposite direction of "group→module feature→module", which meant the sample group difference caused the module feature to change, and finally, the module also changed.

Hence, both models used the feature as the potential mediator and tested the statistical significance of its mediating effect. In addition, the function *diffwgcna* had a parameter *confoundings*, which could be used to transfer potential confounding factors so that their influence on the models could be adjusted during model training. More details about this mediation analysis were provided in Supplementary Data.

### Multi-omics clustering (*multiCCA* and *wgcnacluster*)

The function *multiCCA* performed multi-omics clustering. It first scaled the omics data so all the features had a unified mean of 0 and a standard deviation of 1. If only two omics were used for clustering, the covariance matrix between them would be calculated, and CCA would be used on it, generating the top 30 CC components for each omic, which were then merged into a single matrix. If a third omic existed, this merged matrix would be treated as a single omic, and the third omic was the other. The same process was used to merge them, and if there were also the fourth or other omics, this process would be repeated to combine all of them into one merged matrix. Then, sample clustering would be performed on it via the *k*-means method.

To determine the optimal *k* value for the *k*-means clustering, *multiCCA* accepted multiple candidate *k* values and selected the optimal one by calculating three internal validation indices: the Silhouette, Calinski and Davies-Bouldin.

In addition, our package contained another function, *wgcnacluster*, which could also perform multi-omics clustering and had almost the same steps as *multiCCA*. However, it did not use CCA but a *WGCNA*-based method to generate the merged matrix from the multi-omics data. Then, *k*-means would be used to perform the clustering. Supplementary Data provided more descriptions of the *multiCCA* and *wgcnacluster* frameworks.

### Multi-omics classification (*omicsclassifier* and *pairedensemblepredict*)

Our package could also perform multi-omics classification, depending on the function *omicsclassifier*. It scaled the omics data first and then performed type-Ⅲ ANOVA on each feature to screen the ones with a *P*-value <0.05 to the response variable (sample classes). They would be used for the classification following several steps (Supplementary Figure S1).

For each single-omic dataset, if the parameter *balanceadj* were set as 1, *omicsclassifier* would use a bagging-SMOTE (synthetic minority over-sampling technique) framework to adjust the data distribution and generate 10 balanced base learner datasets. Then, elastic net would be used on them to select the features that could best predict sample classes, and the ones recurrently selected from different base learners would be further used to train 10 SVM (support vector machine) or MLR (multinomial logistic regression) models, also on the base learner datasets, which would be finally aggregated into an ensemble model.

The parameter *balanceadj* could also be set as 2. In this case, *omicsclassifier* would not use the bagging-SMOTE framework. Instead, it would train a normal bagging model using bootstrapping to generate base learner datasets. In addition, if *balanceadj* were set as 3, neither bagging-SMOTE nor bootstrapping would be conducted, and *omicsclassifier* would directly use the original training data to perform the elastic net feature selection and the SVM/MLR model training.

If the data transferred to *omicsclassifier* were single-omic, the above process would return the final classification result. However, if the data were multi-omics, the process would be used on each single-omic first. Then, their predicted sample class results would be aggregated into a final one. Supplementary Data provided more descriptions of the *omicsclassifier* method.

Finally, the classifier trained from *omicsclassifier* could be transferred to another function, *pairedensemblepredict*, which predicted new sample classes with this classifier.

### Network-based gene set function annotation (*diffwgcna*)

The *WGCNA* modules' gene set function annotation could be performed by *diffwgcna*. Unlike traditional enrichment methods that only covered a module's nodes (genes), *diffwgcna* further considered its network structure, reflected by the network edges and their weights.

In our edge-based analysis, the function terms of each edge (gene-gene pair) were defined as the intersection of its two genes'. Then, two methods could be used on them to perform gene set function annotation: (i) edge weight shuffling and (ii) hypergeometric test.

For the edge weight shuffling method, when it was applied to a *WGCNA* module, all the edges there exchanged their weight values mutually and randomly. Hence, each of them would get a new weight value originally belonging to another edge.

Notably, only their weights were shuffled, and their function terms were not. Then, *diffwgcna* focused on the function terms. For each of them, all the edges with this term would be found, and their weights would be summed up and used as this function's weight. Because the edges never exchanged their function terms, after a shuffling, the same edges would be found as those with a specific function. However, these edges' weights were changed during the shuffling, so their weight sum would change, i.e. the function's weight would change after each shuffling.

Such shuffling would be performed 1000 times, so a function would get 1000 different weights, forming a weight distribution. Furthermore, its original weight calculated from the original module edge weights would be mapped to this distribution to get a *P*-value, indicating whether this function's original weight was significantly large. Finally, all the *P*-values calculated from this shuffling method would be adjusted with Benjamini–Hochberg correction.

In addition, *diffwgcna* could also use the other hypergeometric test method to perform gene set annotation, and its details were in Supplementary Data.

### Comparison with other public tools (*iClusterPlus, MOGONET* and *EnrichR*)

For multi-omics or single-omic clustering, the performance of *CWGCNA* was compared with the public tool *iClusterPlus* (23,24). If the dataset contained RNA or miRNA omic, their log_2_(TPM + 1) values were used. If the dataset had DNAm omic, the probe M values were transferred to the function *iClusterPlus* in the R package *iClusterPlus*. In addition, the function's parameter *K* was set from 1 to 5, meaning to cluster the samples into 2 to 6 clusters. Then, the optimal cluster number was selected from them. For other parameters, they were set as their default values.

For multi-omics classification, *CWGCNA* was compared with the *MOGONET* model (25). Before starting the *MOGONET* training, we conducted data preprocessing following its original study.

For *WGCNA* module gene set function annotation, *EnrichR* was used for gene-node-based enrichment (26). The function *enrichr* in the R package *enrichR* accepted the gene names via its parameter *genes*, and the other parameter *databases* was used to specify the background databases for the enrichment analysis. In this study, the GO and Reactome ones were chosen. Correspondingly, the edge-based method in *CWGCNA* was also based on the GO and Reactome databases when using its intersection strategy to get the edge function terms. More details about applying these public tools were described in Supplementary Data.

## Results

### Package overview

**Figure 1.**
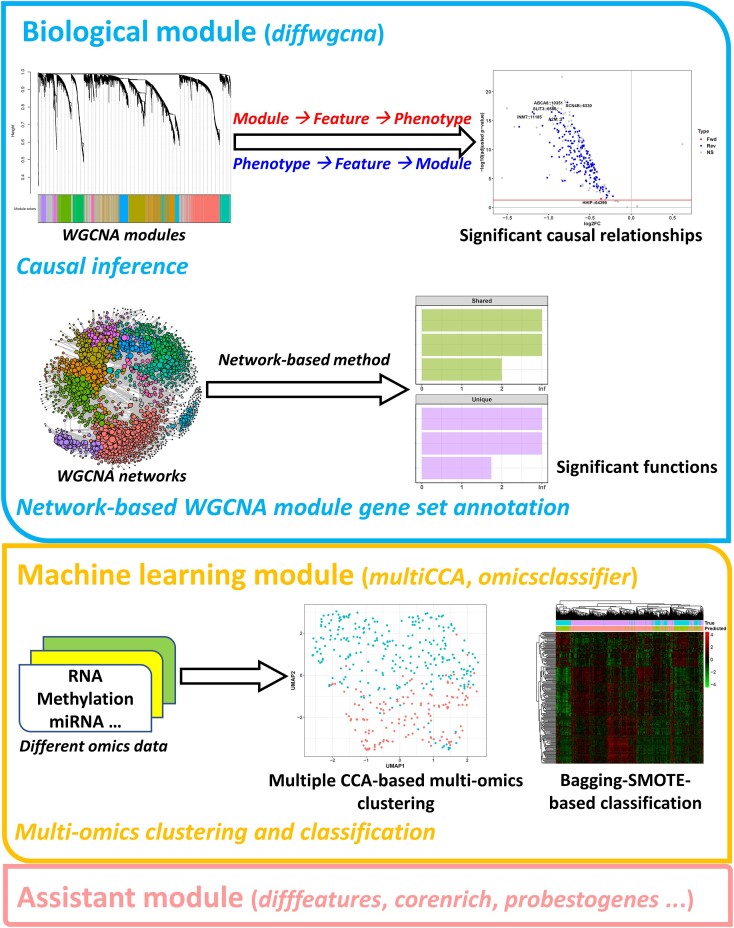
Package modules. The package has three modules: the biological module, the machine learning module, and the assistant module.

The package contains three sections (Figure 1). The first is the biological one. It performs causal *WGCNA* analysis by inserting a mediation inference step into the traditional *WGCNA* pipeline, revealing the causal relationship among phenotypes, *WGCNA* modules, and module features. Considering the potential influence of confounding factors on this analysis, confounding factor identification and adjustment will also be conducted. In addition, when annotating the module gene set functions, this section uses a network-based method, considering the modules' topological structures and capturing their influence on the gene set functions.

The second section is the machine learning one. It clusters samples from multi-omics data based on a multiple CCA method. It also performs multi-omics classification and trains models based on different ensemble frameworks, including a bagging-SMOTE (synthetic minority over-sampling technique) and a bootstrapping framework, or directly constructs models without them. Each model has its unique advantage in overall or rare sample prediction. Moreover, this section is related to *WGCNA* because it can also perform multi-omics clustering via a method based on *WGCNA* modules from different omics.

The final is the assistant section. It provides various functions, such as differential feature identification, methylation site conversion, missing value imputation, etc.

The details can be found in the Materials and Methods part and Supplementary Data.

### The package finds the causal gene of preeclampsia from DNA methylation data and divides this disease into 2 subtypes

To test the performance of *CWGCNA*, we applied it to three single or multi-omics datasets. The single-omic one was a human placenta DNA methylation (DNAm) dataset collected from various studies. After data preprocessing and batch adjustment, we only kept the probes with high quality and were covered by all the Illumina 27K and 450K datasets. The final contained 18 626 probes and 359 samples, including 258 normal samples and 101 disease samples with preeclampsia pregnancy complications caused by placental abnormalities. In addition, the metadata of the 359 samples covered four phenotypic variables, i.e. sample group (preeclampsia or control samples), gestational week, baby gender, and maternal ethnicity.

We first used the biological module of *CWGCNA* to explore the biological rules underlying these data, and we started with our function *featuresampling*, which detected confounding factors by checking the relationship between the DNAm beta values and the metadata. For each phenotypic variable, this function performed type-Ⅲ ANOVA to calculate its averaged *F* statistic across the top 10 000 most variable DNAm probes, representing the variance it explained for the dataset. The result showed that the sample group, gestational week, and baby gender accounted for the most variance (Supplementary Figure S2A). It implied that (i) this dataset was suitable for checking the DNAm difference between the sample groups; (ii) but variance from the gestational week and baby gender needed to be removed. They were confounding factors for preeclampsia analysis.

This conclusion was important to another function, *diffwgcna*, in our package. It performed causal *WGCNA* by inserting a mediation inference step into the traditional pipeline, and the confounding information here was necessary because this analysis assumed that no unmeasured confounding existed for the causal relationship. In the real world, it was impossible to measure and adjust all confoundings, but most of them needed to be processed for good approximation.

**Figure 2.**
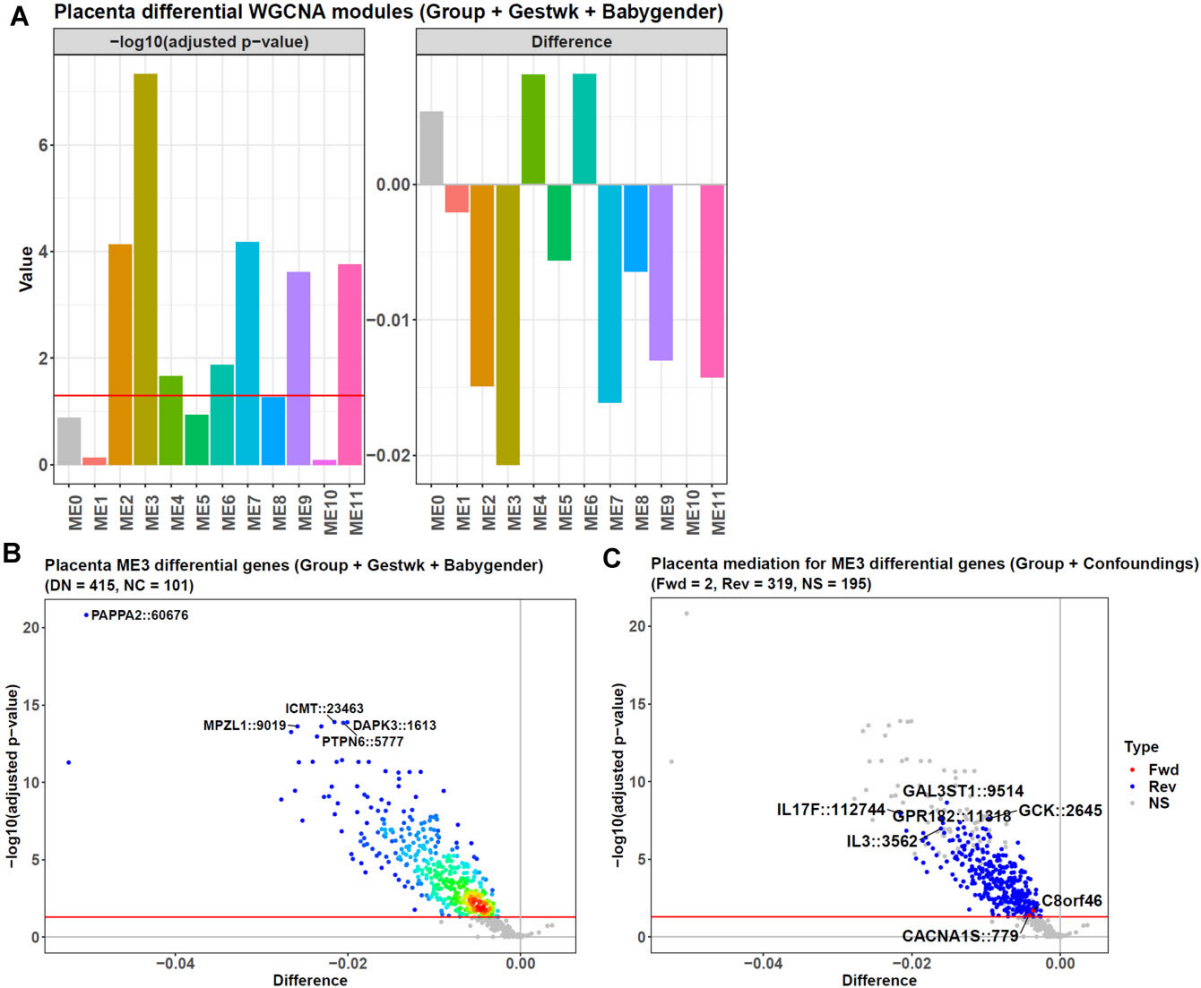
Causal *WGCNA* performance on placenta DNA methylation data. (**A**) The *diffwgcna* function identifies 11 *WGCNA* modules, and seven have largely different eigengenes between the preeclampsia/control groups. (**B**) Within the ME3 module, 415 of its 516 genes show significantly different beta values between the preeclampsia/control groups. The genes with an adjusted *P*-value <0.05 are represented as colorful dots. (**C**) In the ME3 module, two of its genes mediate the relationship of "ME3→ME3 gene→preeclampsia" (the forward direction, red dots), and 319 mediate "preeclampsia→ME3 gene→ME3" (the reverse direction, blue dots). The y-axis and x-axis show the -log_10_(adjusted *P*-value) and preeclampsia/control beta value difference when screening the differential genes before this causal inference.

Then, we used *diffwgcna* on the dataset, and to facilitate the downstream biological explanation, we compressed the DNAm probe beta values to genes in advance. Based on the traditional *WGCNA* pipeline, *diffwgcna* called 11 *WGCNA* modules from the placenta DNAm dataset. Among them, seven were further identified as related to the phenotype, i.e. their eigengenes significantly differed between the preeclampsia and the control samples (Figure 2A). Here, *diffwgcna* utilized *limma* to find the seven modules, different from the traditional method using correlation coefficient. Its advantage was that the *limma* regression could adjust the confounding factors from *featuresampling* and avoid their interference with the result.

Among the seven *WGCNA* models, the module ME3 was the most differential, having the smallest adjusted *P*-value and the largest absolute difference between the 2 groups. Moreover, within ME3, *diffwgcna* further found that 415 of its 516 genes were significantly hypomethylated in preeclampsia samples (Figure 2B), which was also achieved via *limma* regression.

Then, with these 415 genes, mediation inference was used to catch the causal relationship among the phenotype (preeclampsia), the module (ME3), and the module features (the 415 differential genes). As described in the Materials and Methods part and Supplementary Data, it constructed mediation models for each gene in ME3. It tested two causal directions: (i) the reverse direction of "preeclampsia→ME3 gene→ME3", which meant the disease caused the gene hypomethylated, and then the whole gene community, i.e. the ME3 module, changed; and (ii) the forward direction of "ME3→ME3 gene→preeclampsia", which meant the ME3 module changed first, and then it drove the disease via its gene. During these tests, the confounding factors from *featuresampling* were adjusted. The result showed that 319 genes mediated the reverse direction, whereas only two genes mediated the forward direction (Figure 2C).

Then, we looked up these genes' functions and checked whether they supported the inference result. The 319 genes were associated with inflammatory stress in preeclampsia, such as IL3 and IL17F, the top genes found in ME3. Both of them were cytokines for inflammation (27,28). Because these genes' causal direction was from preeclampsia to ME3, the conclusion was that inflammation was the result, but not the cause, of this disease.

Meanwhile, CACNA1S was the top gene mediating the causal direction from ME3 to preeclampsia. This gene had expression in the placenta (29,30), and encoded a calcium channel responsible for blood vascular contraction. Given that preeclampsia was a pregnancy hypertension disease, the driving effect of CACNA1S on it was clear. Furthermore, this gene was the target of amlodipine besylate, a small-molecule drug for preeclampsia treatment, further supporting that the inference here was reasonable. For other differential modules besides ME3, this mediation inference could also be used to detect any other driver or passager genes.

After these biological explorations, we used the machine learning module of *CWGCNA* to perform some machine learning tasks on the dataset. First, we tried to cluster the preeclampsia samples into subtypes with the function *multiCCA*. It used multiple CCA to convert multi-omics data into a compressed one and then performed *k*-means on it to get the sample clustering result. On the single-omic here, the multiple CCA became PCA for the DNAm probe data of the 101 preeclampsia samples.

At the same time, we compared *multiCCA* with a published tool, *iClusterPlus*. It was the enhanced version of *iCluster*, a widely used method to integrate multiple genomics data and cluster the samples using a joint latent variable model (23,24). In addition, it was also applicable to single-omic data.

**Figure 3.**
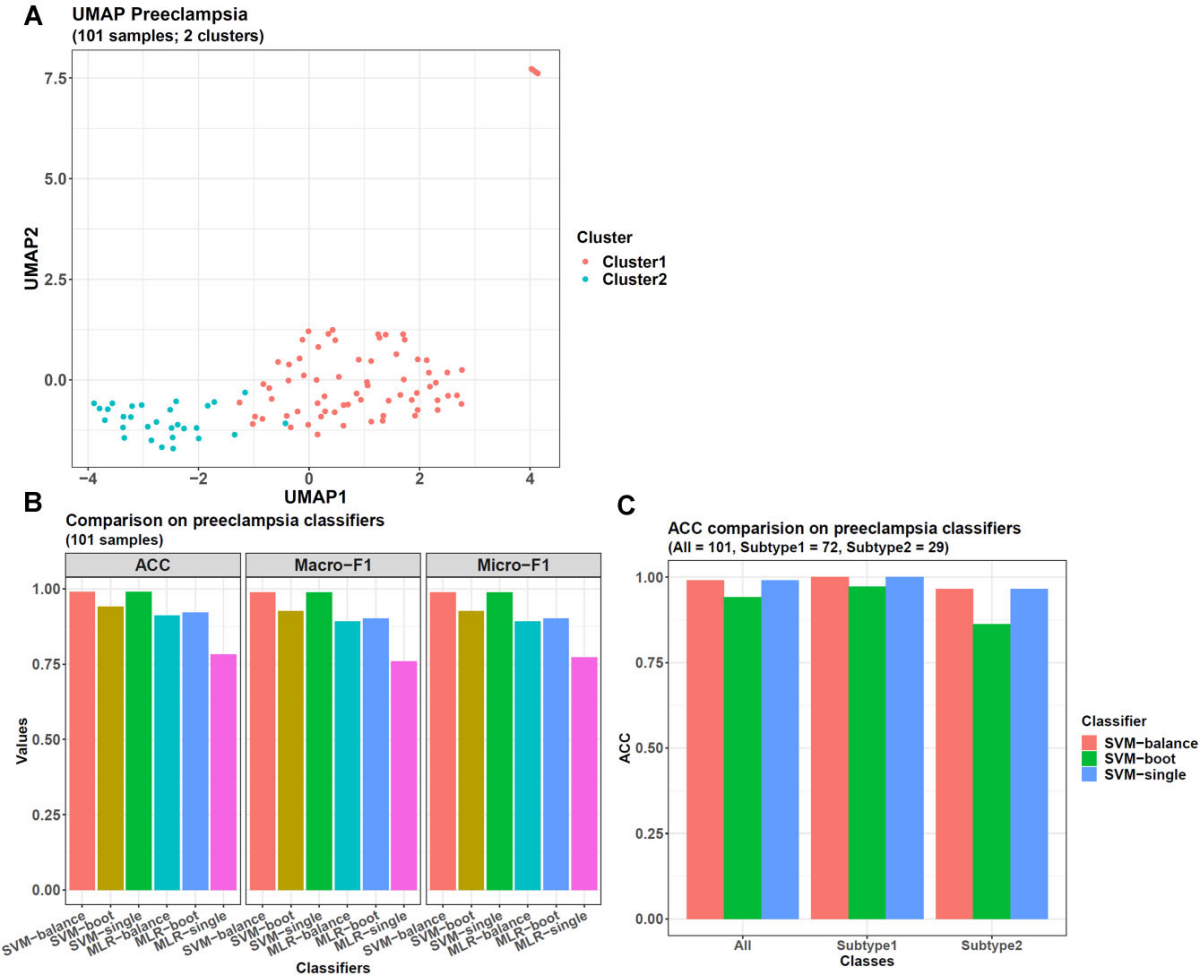
Machine learning results on preeclampsia DNAm data. (**A**) UMAP embedding of the 2 preeclampsia subtypes clustered by *multiCCA* and *iClusterPlus*. (**B**) The 6 *omicsclassifier* models perform differently when classifying preeclampsia subtypes. For the testing samples from all the 5-fold CV sets, the SVM models perform better than the MLR ones. SVM-balance and SVM-single are the most accurate models, with an ACC of 0.99, a macro-F1 of 0.988, and a micro-F1 of 0.988. (**C**) When looking at the SVM models' ACCs on each sample class, SVM-balance and SVM-single show the best ACC (0.966) on subtype2, the smaller sample class.

Finally, *multiCCA* and *iClusterPlus* gave the same result on the dataset here. Both clustered the 101 preeclampsia samples into 2 groups, with the larger one (subtype1) containing 72 samples and the smaller one (subtype2) including 29 (Figure 3A). Moreover, for both methods, this 2-cluster result was better than choosing other cluster numbers from 3 to 6, as shown by the three internal validation indices: the Silhouette, Calinski-Harabasz, and Davis-Bouldin indices. They were calculated from the DNAm probe data based on the clustering results. When the cluster number was 2, *multiCCA* and *iClusterPlus* obtained the maximum Silhouette and Calinski indices and the minimum Davies index (Silhouette = 0.141, Calinski = 19, Davies = 2.05) (Supplementary Figure S3), which meant, in this case, the samples in the same cluster tended to have smaller distance with each other, whereas that in different clusters tended to be farther away from each other.

After getting the subtype labels, we trained machine learning models to classify them. Given that the 2 subtypes had imbalanced sample sizes (72 versus 29 samples), our function *omicsclassifier* was suitable for this task. As described in the Materials and Methods part and Supplementary Data, it had an ensemble framework combining the bagging and the SMOTE methods, which adjusted the imbalanced sample distribution so that the model could be trained from a balanced distribution. This made the final model more accurate in rare sample label prediction (Supplementary Figure S1). In addition to balancing sample distribution, the bagging-SMOTE framework also performed feature selection with elastic net. Then, it used these features to construct several SVM (support vector machine) or MLR (multinomial logistic regression) base learners and finally ensembled their results.

Moreover, *omicsclassifier*'s ensemble framework was not limited to the bagging-SMOTE one; it could also use a bootstrapping framework without any distribution adjustment or skip the frameworks and only perform the feature selection and SVM/MLR model training. These methods could be used for both single-omic and multi-omics data.

Here, we trained all 6 models provided by *omicsclassifier*, i.e. the SVM and MLR models combined with the bagging-SMOTE framework (the SVM-balance and MLR-balance models), the bootstrapping framework (the SVM-boot and MLR-boot models), and without any framework (the SVM-single and MLR-single models). Then, we checked their performance on the preeclampsia subtype dataset with a 5-fold cross-validation (5-fold CV).

On the CV's testing set, whose data distribution was not influenced by any model's training framework, all the SVM models performed better than the MLR ones (Figure 3B), showing higher accuracy (ACC), macro-F1, and micro-F1 values. Among them, the SVM-balance and SVM-single models were the best. They misclassified only one sample, so both had an ACC of 0.99, a macro-F1 of 0.988, and a micro-F1 of 0.988.

Furthermore, we checked the SVM models' ACCs on each sample class (Figure 3C). The sample misclassified by SVM-balance was the same as SVM-single. It was from the small class (a subtype2 sample), so both the two models had an ACC of 0.966 on this class, higher than SVM-boot with an ACC of 0.862 here. Hence, SVM-balance and SVM-single obtained the best ACC on small class samples.

This case study showed the performance of *CWGCNA* on single-omic data. In addition to the causal *WGCNA* and machine learning tasks, our package could perform other basic analyses, such as differential feature identification, gene functional enrichment, DNAm probe to gene conversion, etc. Hence, we used them to explore the biological meaningness of the 2 preeclampsia subtypes. We checked the differential DNAm probes between the subtypes and found 674 hyper and 12 865 hypomethylated ones in subtype2 relative to subtype1 (Supplementary Figure S4A). After that, we compressed these probes to genes and functional enrichment found that the hypomethylated genes were associated with immune functions, such as "T cell receptor regulation of apoptosis", indicating subtype2's close relationship with the inflammatory stress of preeclampsia (Supplementary Figure S4B). On the other hand, the hypermethylated genes in subtype2, i.e. the hypomethylated ones in subtype1, were enriched in "Oncostatin M" and "Statin pathway", which had also been reported as highly related to this disease (31,32).

### The package finds 2 lung cancer subtypes from multi-omics data and identifies the causal gene for the subtype divergence

Next, we applied the package to the TCGA LUAD (lung adenocarcinoma) dataset, which contained 419 cancer samples and three different omics: RNA, DNAm (450K) and miRNA. After preprocessing, their feature numbers were 21 465 RNA genes, 412 481 DNAm probes and 1574 miRNA genes, respectively.

Then, we selected the top 12 000 most variable genes and probes from the RNA and DNAm datasets, respectively. Together with the 1574 miRNA genes, they were transferred to *multiCCA* and *iClusterPlus* to compare their clustering on the samples. In addition, our package had another function, *wgcnacluster*, that could be used for clustering in a multi-omics situation. It performed *WGCNA* on each omic and then combined their *WGCNA* eigengenes for the sample clustering.

The result showed that both *multiCCA* and *wgcnacluster* had an optimal cluster number of 2 because their integrated data matrices obtained the maximum Silhouette and Calinski indices in this case (*multiCCA*'s Silhouette = 0.126 and Calinski = 59.2; *wgcnacluster*'s Silhouette = 0.123 and Calinski = 60.2), much larger than other cluster numbers from 3 to 6 (Supplementary Figure S5). However, these indices could not be compared between the two methods to judge which was better because they were calculated from different integrated data matrices; one was generated via multiple CCA and the other via *WGCNA*. Hence, we changed to survival analysis and compared the methods' inter-cluster survival differences.

**Figure 4.**
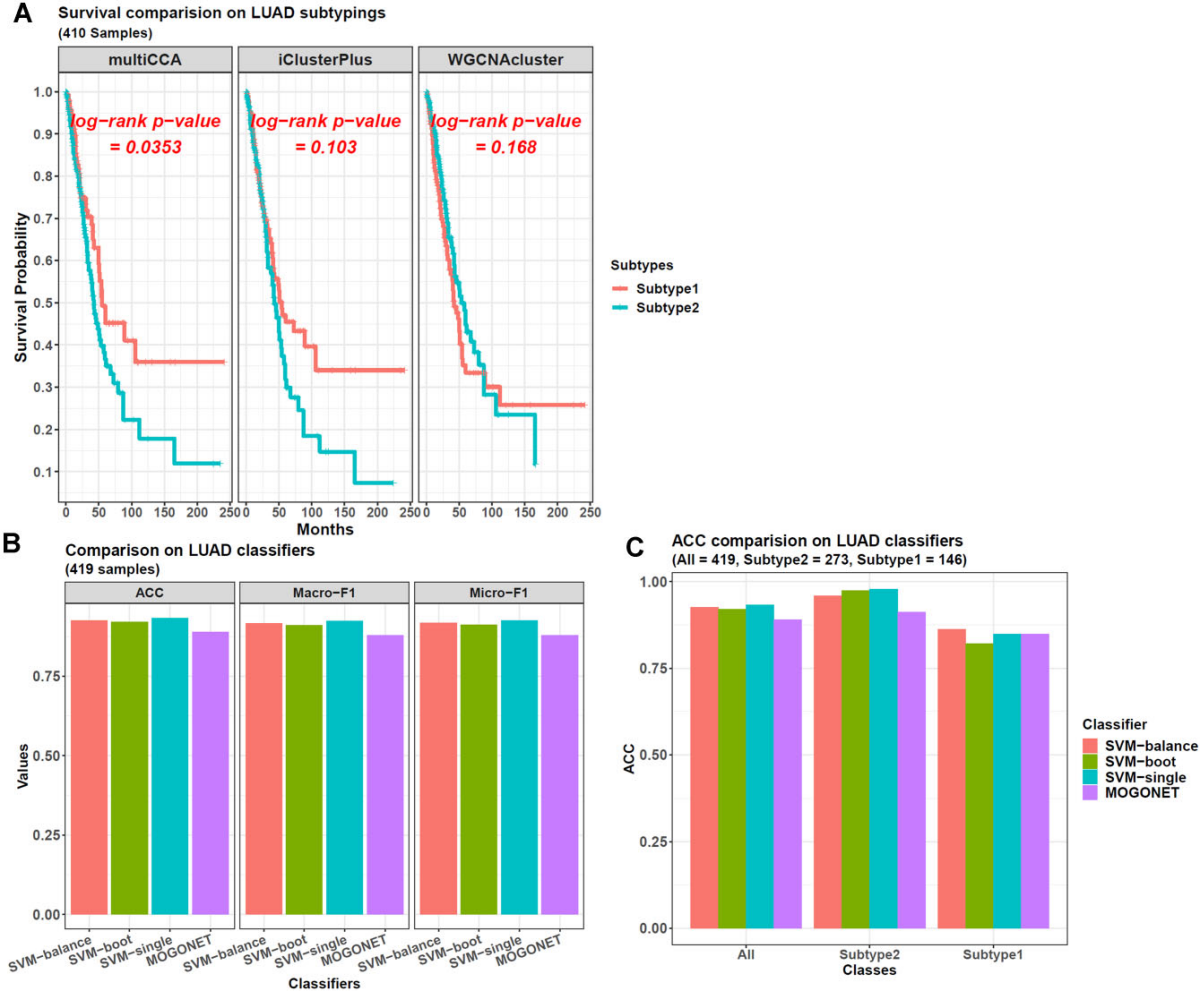
Machine learning results on LUAD multi-omics data. (**A**) The 2 LUAD subtypes clustered by *multiCCA* show a significant difference in sample survival time, better than that from *iClusterPlus* and *wgcnacluster*, which do not have this significance. Because only 410 samples of the original 419 ones have survival information, only they are included here. (**B**) The 3 SVM models from *omicsclassifier* perform better than *MOGONET* in classifying LUAD subtypes. For the testing samples from all the 5-fold CV sets, the models' performance rank is SVM-single > SVM-balance > SVM-boot > *MOGONET*. SVM-single has the highest ACC of 0.933, macro-F1 of 0.924, and micro-F1 of 0.926. In contrast, *MOGONET*'s performance is weaker than others, with an ACC of 0.89, a macro-F1 of 0.88, and a micro-F1 of 0.88. (**C**) When looking at the models' ACCs on each sample class, SVM-balance shows the best ACC (0.863) on subtype1, the smaller sample class.

The result showed that only *multiCCA*'s clusters had a significant sample survival difference, and the log-rank *P*-value was 0.0353, whereas that of *iClusterPlus* had a *P*-value of 0.103 and that of *wgcnacluster* was 0.168, weaker than *multiCCA* (Figure 4A).

It was noteworthy that *iClusterPlus*'s optimal cluster number was not 2 but 6 because its internally calculated BIC (Bayesian information criterion) value reached the minimum when the cluster number was 6 (BIC = 8 739 347) (Supplementary Figure S6A). Hence, we also checked the survival difference among these 6 sample groups, and the log-rank *P*-value was 0.0651 (Supplementary Figure S6B), still weaker than *multiCCA*'s 0.0353.

Hence, *multiCCA* performed better, and we chose its 2 clusters for downstream analysis. The first cluster (subtype1) contained 146 samples with a significantly longer survival time, and the second cluster (subtype2) had 273 samples.

Then, we trained the *omicsclassifier* models with these subtypes as true labels. We only included the 3 SVM models here because of their better performance in the placenta study. This time, they were trained from the RNA/DNAm/miRNA multi-omics data. In addition, we introduced another published tool, *MOGONET*, to this task. It was a graph convolutional network (GCN) specially developed for multi-omics classification and was reported as better than other methods (25). We wanted to compare its performance with *omicsclassifier*.

For *MOGONET*, we trained it following its original study, using all the same parameters and screening the multi-omics features for it with an ANOVA method. For *omicsclassifier*, it automatically prescreened the features before other formal steps, also based on ANOVA. Hence, these two methods used similar features as initial.

Finally, the 5-fold CV showed that the *omicsclassifier* models were better than *MOGONET* (Figure 4B). Their performance rank was SVM-single > SVM-balance > SVM-boot > *MOGONET*. SVM-single had the highest ACC of 0.933, macro-F1 of 0.924, and micro-F1 of 0.926. In contrast, *MOGONET*'s performance was weaker than others, with an ACC of 0.89, a macro-F1 of 0.88, and a micro-F1 of 0.88.

Then, we further checked the models' ACCs on each sample class. This time, subtype1 had 146 samples, about half of subtype2's 273 samples. This smaller sample size made all the models perform weaker, with SVM-balance performing relatively well. Its ACC was 0.863, better than others and showing the effect of its bagging-SMOTE framework in improving rare sample prediction (Figure 4C).

After these machine learning tasks, we switched to the biological module of *CWGCNA* and explored the subtypes' biological properties. We still started from the function *featuresampling* to get the confounding information. This LUAD dataset had five phenotypic variables. Besides the sample subtypes from *multiCCA*, there were four confounding factors from TCGA, including patients' gender, age, ethnicity and cigarettes used per day. They all had an *F* statistic >1 on the RNA data, according to *featuresampling*'s ANOVA result (Supplementary Figure S2B).

**Figure 5.**
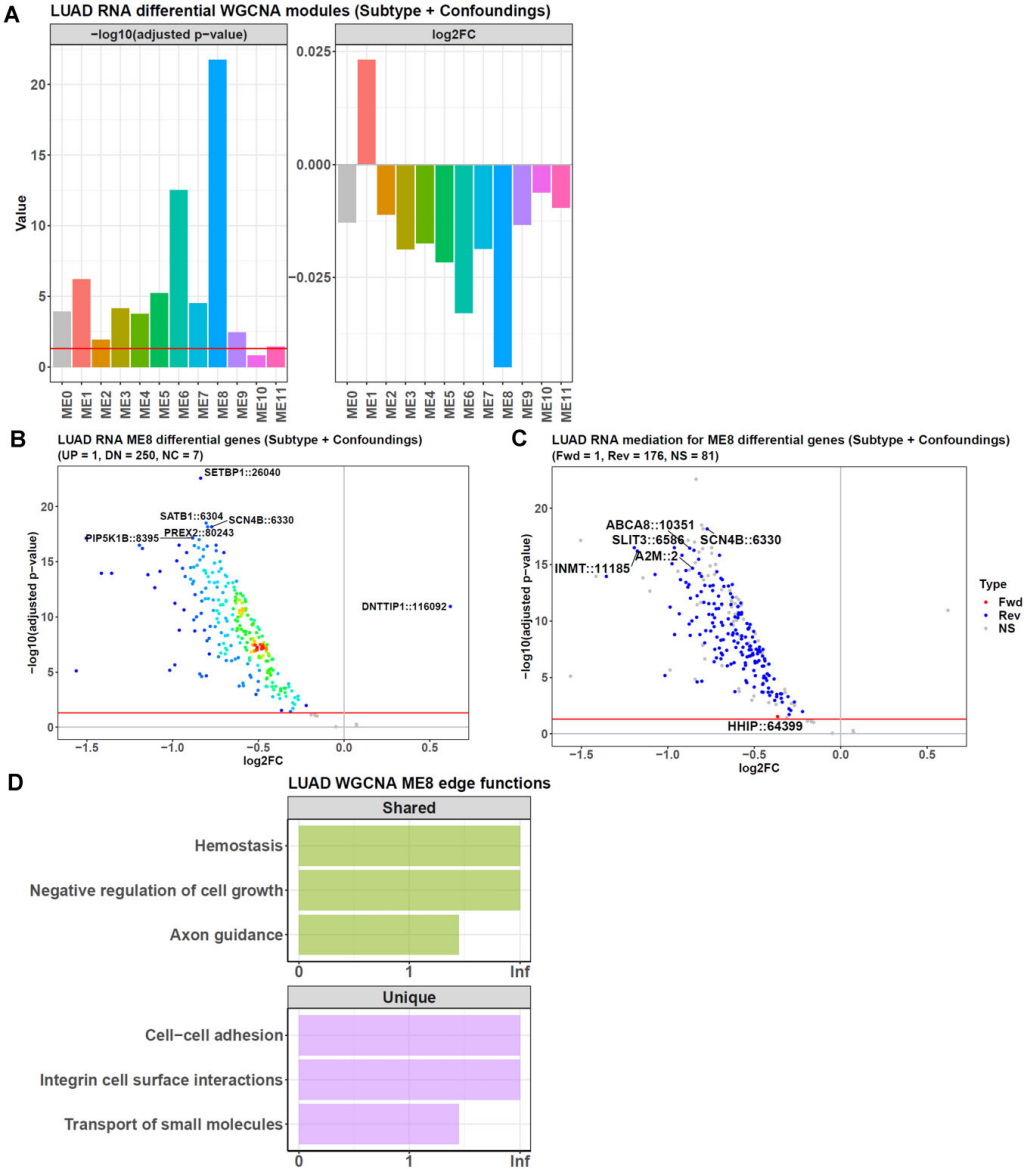
Causal *WGCNA* performance on LUAD RNA data. (**A**) The function *diffwgcna* detects 11 modules in the LUAD RNA dataset, and 10 have significantly different eigengenes between the subtype2/subtype1 groups. (**B**) Within the ME8 module, 251 genes have significantly different expressions between the 2 LUAD subtypes. (**C**) Mediation analysis finds one gene in ME8 mediates the causal direction of "ME8→ME8 gene→LUAD subtypes" (the forward direction, red dot), and 176 mediate "LUAD subtypes→ME8 gene→ME8" (the reverse direction, blue dots). The y-axis and x-axis show the −log_10_(adjusted *P*-value) and subtype2/subtype1 RNA log_2_FC difference when screening the differential genes before this causal inference. (**D**) Edge-based function annotation for the gene set in ME8. The green bars are functions identified by both the edge-based method and *EnrichR*, and the purple bars are only found as significant by the edge method. The x-axis represents −log_10_(adjusted *P*-value) in the edge-based method. *EnrichR* uses the GO and Reactome databases as its enrichment background, whereas the edge method performs its intersection strategy on these databases to get the edge function terms for the downstream shuffling step.

Then, we performed causal *WGCNA* with these confoundings adjusted. The *diffwgcna* function detected 11 modules in the RNA data, and 10 of them showed a significant eigengene difference between the subtypes (Figure 5A). The module ME8 was the most differential, and further within-module detection found it had 250 genes down-regulated and one gene up-regulated in subtype2 relative to subtype1 (Figure 5B).

Then, the causal inference was applied to ME8 and these 251 genes, with two causal directions tested: (i) in the direction of "ME8→ME8 gene→LUAD subtypes", only one gene was detected as significant, indicating it could drive the LUAD subtype divergence, (ii) in the opposite direction of "LUAD subtypes→ME8 gene→ME8", 176 genes were identified as significant, meaning they were passager genes and they changed expression following the subtype progress (Figure 5C).

A deep look at these genes supported the reasonableness of the inference. For the one driver gene, it was HHIP, a hedgehog-interacting protein associated with cancer stemness (33,34). Given cancer stem cells' tumor-initiating capacity, it was understandable that HHIP drove the LUAD subtype development here.

In contrast, the 176 passager genes were biased toward functions developed with cancer progress, such as their top genes, SCN4B and SLIT3, which controlled cancer cell migration (35,36), and the ABCA8 gene that related to cancer chemoresistance (37).

After the causal inference, we used *diffwgcna* to perform network-based gene set function analysis. It was specifically designed for *WGCNA* modules. In this case, each module was seen as a gene network, and when annotating its gene set function, *diffwgcna* used the network edges' function, rather than the gene nodes', to perform functional enrichment. This was because if only using the gene nodes, any modules with the same nodes would have the same function, even if their network structures, i.e. the network edges and edge weights, were different. For the edges' function, because an edge was a gene-gene pair, *diffwgcna* used these two genes' intersection as the edge's function. Then, an edge weight shuffling method was used to find the functions with significantly large weights.

Briefly, when shuffling on a *WGCNA* module, all its edges mutually and randomly exchanged their weight values, so after that, each of them got a new weight value originally belonging to another edge. Notably, only their weights were shuffled, and their function terms were not. Then, for a specific function term, all the edges with it would be found, and their weights' sum would be used as this function's weight. Because the edges' weights changed with the shuffling, a function term would get 1000 different weights after 1000 times of shuffling, forming a weight distribution. Furthermore, its original weight from the original module edge weights would be mapped to this distribution to get a *P*-value, indicating whether this function's original weight was significantly large.

For the module ME8 here, its function was analyzed by this edge-based method. At the same time, we used *EnrichR* to perform gene-node-based enrichment on it (26). The result showed that some functions were significant in both methods, such as "Negative regulation of cell growth" (Figure 5D). Meanwhile, the edge-based method also found some unique functions, including "Cell-cell adhesion" and "Integrin cell surface interactions". The edges with these functions had unusually large weights, so their functions were identified by the edge method.

These cell adhesion functions were obviously related to cancer development, including LUAD. They normally participated in various cellular activities, such as embryogenesis, cell migration, proliferation, etc. In contrast, their alterations in cancer promoted uncontrolled cell growth and progressive distortion of tissue architecture, making cells gain a more motile and invasive phenotype (38).

When performing the *EnrichR* gene functional analysis above, we chose the GO and Reactome databases as enrichment backgrounds. Correspondingly, the edge-based method used its intersection strategy to get the edge function terms, also from these backgrounds. So, the two methods were based on the same databases.

This case study showed the application of *CWGCNA* in multi-omics data analysis. For the 2 LUAD subtypes, we also tried to understand the reason for their different survival times. Hence, we used the basic functions in our package to check their differential genes from the RNA data and found that the LUAD subtype2 samples had 1522 up-regulated and 8663 down-regulated genes relative to subtype1 (Supplementary Figure S4C). Further functional enrichment showed that the up-regulated genes were associated with some tumor-promoting functions, including "Cell cycle", "DNA replication", etc. In contrast, tumor-suppressive functions such as the "T cell receptor/JNK pathway" and "Wnt signaling pathway" were down-regulated (Supplementary Figure S4D). This explained the observation that subtype2 samples had a much weaker survival status than subtype1 (Figure 4A).

These differential feature identification, functional enrichment, and other basic analysis tasks could be achieved directly with our package via the functions *difffeatures*, *corenrich*, etc.

### The package finds the causal gene for non-Luminal A/Luminal A breast cancer subtype divergence and trains accurate subtype classifiers from multi-omics data

We also tested the package using 752 cancer samples in the TCGA BRCA (breast invasive carcinoma) dataset, covering RNA, 450K DNAm, and miRNA omics. This time, we compressed the DNAm probe values to genes to see the package performance on DNAm features other than probes. After preprocessing, the feature numbers were 21 502 RNA genes, 18 648 DNAm genes and 1582 miRNA genes. In addition, we collected the BRCA subtype information from *TCGAbiolinks* (19), which labeled the samples as 5 PAM50 subtypes (411 Luminal A samples, 137 Luminal B samples, 126 basal-like samples, 44 HER2-enriched samples and 34 normal-like samples).

**Figure 6.**
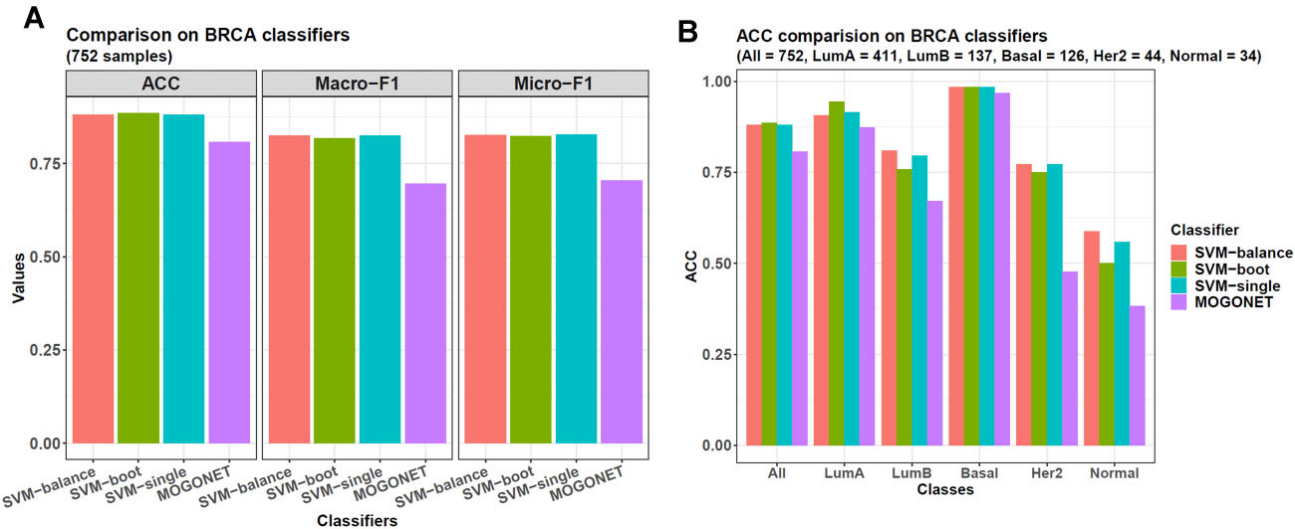
Machine learning results on BRCA multi-omics data. (**A**) The 3 SVM models from *omicsclassifier* perform better than *MOGONET* in classifying the 5 BRCA subtypes. For the testing samples from all the 5-fold CV sets, the SVM-boot model has the highest ACC of 0.886, and the SVM-single model has the largest macro-F1 of 0.824 and micro-F1 of 0.828. In contrast, *MOGONET*'s performance is weaker than others, with an ACC of 0.807, a macro-F1 of 0.695, and a micro-F1 of 0.705. (**B**) When looking at the models' ACCs on each sample class, SVM-balance performs best on the small classes, with an ACC of 0.773 on the HER2 samples and 0.588 on the normal-like samples.

Because we already had the subtype labels this time, we directly used *omicsclassifier* to train models from the three omics. For the RNA and DNAm data, their top 10 000 most variable genes were used, and for the miRNA data, all the 1582 miRNA genes were used. We also included *MOGONET* here to compare it with *omicsclassifier*'s SVM models. Then, the 5-fold CV showed that *MOGONET* was still weaker than *omicsclassifier* (Figure 6A). Its ACC, macro-F1, and micro-F1 were all weaker than other models (ACC = 0.807, macro-F1 = 0.695, and micro-F1 = 0.705). On the other hand, each of the SVM models had a unique advantage. The SVM-boot model had the largest ACC of 0.886, whereas the SVM-single model had the highest macro-F1 of 0.824 and the highest micro-F1 of 0.828. On the other hand, if looking at the ACCs for each sample class, SVM-balance showed its advantage (Figure 6B). The HER2-enriched and normal-like subtypes only contained 44 and 34 samples, respectively, making all the models perform weak on them. However, SVM-balance was relatively good. Its ACC on the HER2 samples was 0.773, and that on the normal-like samples was 0.588, already better than other models. Hence, SVM-balance predicted rare samples best in all the placenta, LUAD and BRCA datasets, which could be attributed to its bagging-SMOTE framework for model training.

Next, we used *CWGCNA*'s biological module to mine more information from this dataset. In addition to the sample subtypes, we obtained two phenotypic variables from TCGA, patients' ethnicity and age, and *featuresampling* found both of them had an *F* statistic >1 on the RNA data (Supplementary Figure S2C), which would be adjusted for the causal *WGCNA* inference.

It should be clarified that this inference was based on mediation models, which worked on data with a continuous or binary response variable. However, the BRCA samples here had 5 subtypes rather than 2. Hence, we kept the 411 Luminal A samples as they were and combined all the other 341 samples into a non-Luminal A group so that the response variable became binary and causal *WGCNA* could be used.

**Figure 7.**
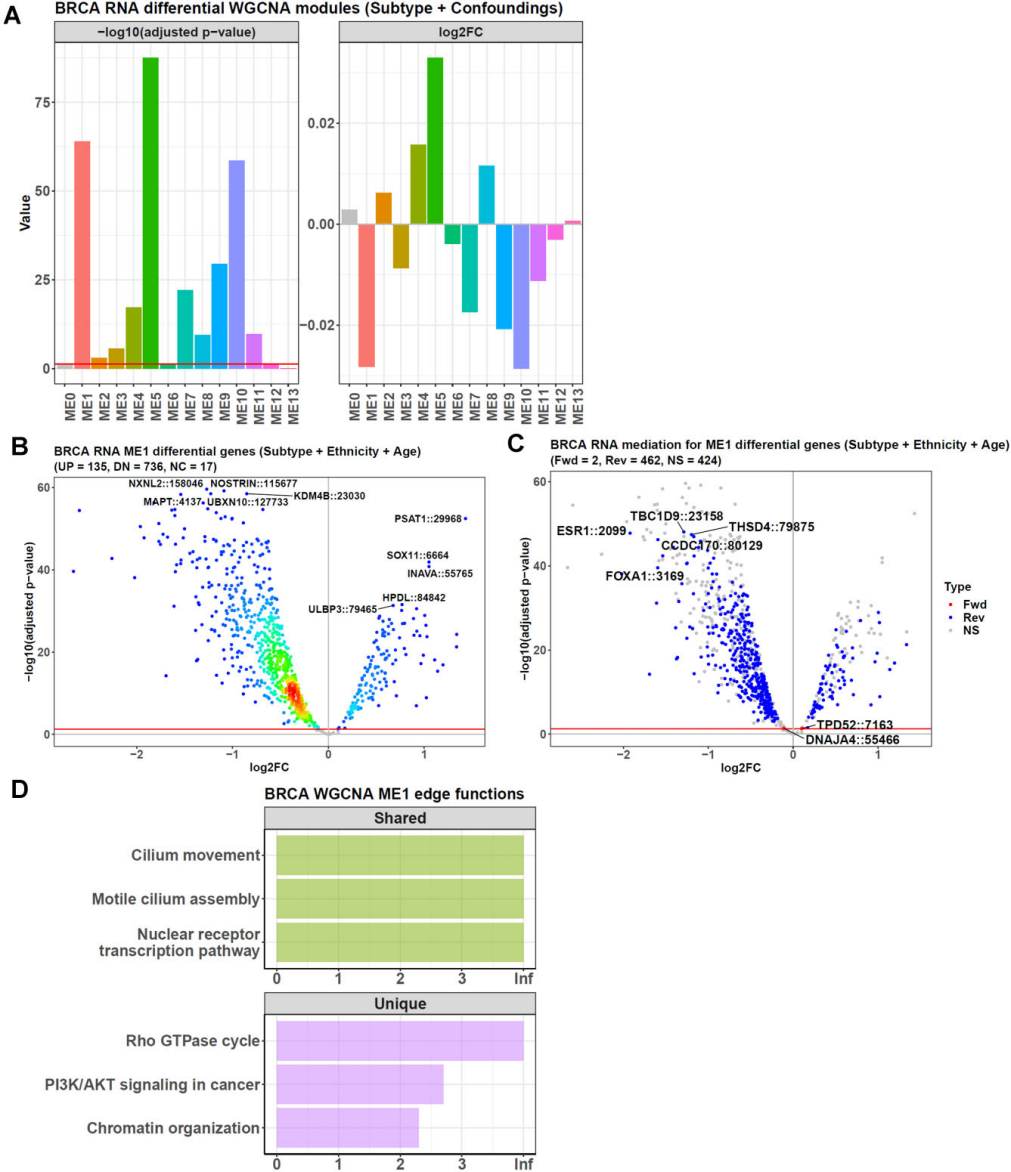
Causal *WGCNA* performance on BRCA RNA data. (**A**) The function *diffwgcna* finds 13 modules in the BRCA RNA dataset, and 11 have significantly different eigengenes between the non-Luminal A/Luminal A groups. (**B**) Within the ME1 module, 871 genes have significantly different expressions between the non-Luminal A and Luminal A samples. (**C**) Mediation analysis finds two genes in ME1 mediates the causal direction of "ME1→ME1 gene→non-Luminal A/Luminal A subtypes" (the forward direction, red dots), and 462 mediate the direction of "non-Luminal A/Luminal A subtypes→ME1 gene→ME1" (the reverse direction, blue dots). The y-axis and x-axis show the −log_10_(adjusted *P*-value) and the non-Luminal A/Luminal A RNA log_2_FC difference when screening the differential genes. (**D**) Edge-based function annotation for the gene set in ME1. The green bars are functions identified by both the edge-based method and *EnrichR*, and the purple bars are functions only found as significant by the edge-based method. The x-axis represents −log_10_(*P*-value) in the edge-based method. *EnrichR* uses the GO and Reactome databases as its enrichment background, whereas the edge method performs its intersection strategy on these databases to get the edge function terms for the following shuffling step.

This time, *diffwgcna* found 13 modules in the RNA data, and 11 had a significant eigengene difference between the non-Luminal A and Luminal A subtypes (Figure 7A). The most differential one was the module ME5. However, among its 373 genes, the causal inference only detected 156 passengers, and no driver gene was found. Hence, we turned to ME1, the second most differential module, because it had two genes identified as drivers.

In total, ME1 contained 888 genes, and 135 were up-regulated in the non-Luminal A samples relative to the Luminal A ones, whereas 736 genes were down-regulated (Figure 7B). The causal inference found two driver genes from them, mediating the direction of "ME1→ME1 gene→ non-Luminal A/Luminal A subtypes". On the other hand, 462 genes were identified as passengers mediating the opposite direction of "non-Luminal A/Luminal A subtypes→ME1 gene→ME1" (Figure 7C).

It was noteworthy that ESR1, the estrogen receptor gene, was found as a passenger rather than a driver here, different from the reports that this gene drove ESR-positive breast cancer, such as Luminal A, to acquire resistance to endocrine therapy (39,40). This inconsistency was because the non-Luminal A group here contained many Luminal B samples (137 samples/341 samples = 40.2%), which were also ESR-positive breast cancer. Hence, for the non-Luminal A/Luminal A divergence analyzed here, ESR1 was not a driver. The same applied to its interacting genes, such as CCDC170 and FOXA1 identified here.

In contrast, the driver genes for this subtype divergence were DNAJA4 and TPD52. For DNAJA4, it correlated to patients survival only in Luminal A samples, whereas the non-Luminal A ones, including Luminal B, did not have this correlation (41), which hinted this gene's potential to drive the non-Luminal A/Luminal A divergence.

Finally, we annotated the ME1 module's function via the edge-based method and *EnrichR*, respectively. This time, both found the function "Nuclear receptor transcription pathway" significant (Figure 7D), showing the close relationship between ME1 and nuclear receptors.

However, the edge-based method also found pathways with significantly large edge weights, such as "PI3K/AKT signaling in cancer" and "Rho GTPase cycle". Correspondingly, many studies showed the PI3K/AKT pathway's close relationship to breast cancer (42–45). It was the most altered pathway in the Luminal A subtype, where the PI3K activating mutation rate was up to 42%, much higher than other subtypes (45). On the other hand, the "Rho GTPase cycle" function was also reported to be important to breast cancer progression, which integrated upstream signals from the tyrosine-kinase and G-protein receptors in Luminal A samples (46).

This case study showed the performance of *CWGCNA* on another multi-omics dataset.

## Discussion

Mining causal pathways from omics data is challenging, and mediation analysis may be useful because it can assess the extent to which some intermediate mechanisms mediate one variable's effect on another (5). However, it has been used minimally in omics analysis. Hence, we developed the package *CWGCNA* to fill this gap.

*CWGCNA* combines mediation analysis with the traditional *WGCNA* framework. It uses the *WGCNA* module, the module features, and the phenotypic traits to construct mediation models and identify a specific phenotype's driver and passenger genes. Our case studies have shown its ability to establish pathological causality in this way.

On the other hand, it is noteworthy that these models assume no unmeasured confounding exists for the causal relationship, and violations can give rise to misleading (6,9,20). Hence, we emphasized confounding adjustment in all the case studies, and our function *featuresampling* can help to determine the datasets' confounding factors.

However, there is always a possibility of unmeasured confounding. In this situation, biological knowledge becomes vital in judging the computational results. For instance, in the LUAD subtype study, although the mediation model adjusted several confoundings, whether there were still missed ones was unknown, making its results not completely confirmed. However, from the genes' biological functions, the identified HHIP gene was closely related to cancer stem cells, which were key drivers of tumor progression (47), so the inference that HHIP caused the subtype divergence became reasonable. It can be seen that biological knowledge coordinates the difficulty of covering all confounding factors in practice. Hence, it should get enough attention when implementing the mediation analysis.

Despite these limitations and assumptions, *CWGCNA* has many strengths. To our knowledge, it is the first package that attempts to detect the causal associations from omics data and the *WGCNA* framework. Many studies examine relationships between diseases and genes, but they are limited by the inability to establish causality. Although *CWGCNA* does not concretely establish a pathway, it provides a further step to the traditional *WGCNA* pipeline and may be particularly useful for omics studies attempting to identify biological mechanisms.

After the causal inference, our package annotates *WGCNA* modules' gene set function with a network-based method. It first gets the function terms of each module edge by intersecting its two genes. Then, it finds the functions with unusually large edge weights. This process considers a module's topological structure and captures its influence on the gene set functions, avoiding the situation that any modules with the same gene nodes will have the same function annotation, even if their structures are different. When applying it to our case studies, we saw some functions that other methods could not find, such as "Cell-cell adhesion" and "Integrin cell surface interactions" in the LUAD case study. These cell adhesion functions are obviously related to cancer development, including LUAD (38).

In addition to conducting these biological explorations, *CWGCNA* also contains a machine learning section. It clusters samples from multi-omics data based on a multiple CCA method. Our case studies have shown that it can find sample clusters with more significant differences than other methods. Besides clustering, our package can also perform multi-omics classification and train various classifiers, which have advantages in different tasks. If focusing on overall sample prediction, the SVM-single model is recommended because it always performs better than others. However, if looking at small class samples, the SVM-balance model should be chosen. Its internal bagging-SMOTE framework can balance the training data distribution and increase the weights of small class samples during training, making this model perform better on rare sample label prediction.

In summary, we developed the R package *CWGCNA*, which introduces mediation analysis into the traditional *WGCNA* framework to conduct causal inference and also perform other novel functions. It provides more possibilities for high-throughput omics data analysis.

## Supplementary Material

lqae042_Supplemental_Files

## Data Availability

*CWGCNA* is available on GitHub (https://github.com/yuabrahamliu/CWGCNA) and Zenodo (https://doi.org/10.5281/zenodo.10982851). Its tutorial can be found in the supplementary files.

## References

[1] Zhang B., Horvath S. A general framework for weighted gene co-expression network analysis. Statist. Appl. Genet. Mol. Biol. 2005; 4:Article17. 10.2202/1544-6115.1128 16646834

[2] Zhou J., Guo H., Liu L., Hao S., Guo Z., Zhang F., Gao Y., Wang Z., Zhang W. Construction of co-expression modules related to survival by WGCNA and identification of potential prognostic biomarkers in glioblastoma. J. Cell. Mol. Med. 2021; 25:1633–1644. 33449451 10.1111/jcmm.16264 PMC7875936

[3] Zuo Z., Shen J.-X., Pan Y., Pu J., Li Y.-G., Shao X.-h., Wang W.-P. Weighted gene correlation network analysis (WGCNA) detected loss of MAGI2 promotes chronic kidney disease (CKD) by podocyte damage. Cell. Physiol. Biochem. 2018; 51:244–261. 30448842 10.1159/000495205

[4] Lin C.-T., Xu T., Xing S.-L., Zhao L., Sun R.-Z., Liu Y., Moore J.P., Deng X. Weighted gene co-expression network analysis (WGCNA) reveals the hub role of protein ubiquitination in the acquisition of desiccation tolerance in Boea hygrometrica. Plant Cell Physiol. 2019; 60:2707–2719. 31410481 10.1093/pcp/pcz160

[5] VanderWeele T.J., Vansteelandt S. Conceptual issues concerning mediation, interventions and composition. Statist. Interface. 2009; 2:457–468.

[6] VanderWeele T.J. Mediation analysis: a practitioner's guide. Annu. Rev. Public Health. 2016; 37:17–32. 26653405 10.1146/annurev-publhealth-032315-021402

[7] Shrout P.E., Bolger N. Mediation in experimental and nonexperimental studies: new procedures and recommendations. Psychol. Methods. 2002; 7:422–445. 12530702

[8] Pearl J. Interpretation and identification of causal mediation. Psychol. Methods. 2014; 19:459–481. 24885338 10.1037/a0036434

[9] Ferguson K.K., Chen Y.-H., VanderWeele T.J., McElrath T.F., Meeker J.D., Mukherjee B. Mediation of the relationship between maternal phthalate exposure and preterm birth by oxidative stress with repeated measurements across pregnancy. Environ. Health Perspect. 2017; 125:488–494. 27352406 10.1289/EHP282 PMC5332184

[10] Novakovic B., Yuen R.K., Gordon L., Penaherrera M.S., Sharkey A., Moffett A., Craig J.M., Robinson W.P., Saffery R. Evidence for widespread changes in promoter methylation profile in human placenta in response to increasing gestational age and environmental/stochastic factors. BMC Genomics. 2011; 12:529. 22032438 10.1186/1471-2164-12-529 PMC3216976

[11] Chu T., Bunce K., Shaw P., Shridhar V., Althouse A., Hubel C., Peters D Comprehensive analysis of preeclampsia-associated DNA methylation in the placenta. PLoS One. 2014; 9:e107318. 25247495 10.1371/journal.pone.0107318 PMC4172433

[12] Hanna C.W., Peñaherrera M.S., Saadeh H., Andrews S., McFadden D.E., Kelsey G., Robinson W.P. Pervasive polymorphic imprinted methylation in the human placenta. Genome Res. 2016; 26:756–767. 26769960 10.1101/gr.196139.115 PMC4889973

[13] Price E.M., Peñaherrera M.S., Portales-Casamar E., Pavlidis P., Van Allen M.I., McFadden D.E., Robinson W.P. Profiling placental and fetal DNA methylation in human neural tube defects. Epigenetics Chromatin. 2016; 9:6. 26889207 10.1186/s13072-016-0054-8 PMC4756451

[14] Leavey K., Wilson S.L., Bainbridge S.A., Robinson W.P., Cox B.J. Epigenetic regulation of placental gene expression in transcriptional subtypes of preeclampsia. Clin Epigenetics. 2018; 10:28. 29507646 10.1186/s13148-018-0463-6 PMC5833042

[15] Wilson S.L., Leavey K., Cox B.J., Robinson W.P. Mining DNA methylation alterations towards a classification of placental pathologies. Hum. Mol. Genet. 2018; 27:135–146. 29092053 10.1093/hmg/ddx391 PMC5886226

[16] Zhou W., Triche T.J. Jr, Laird P.W., Shen H SeSAMe: reducing artifactual detection of DNA methylation by Infinium BeadChips in genomic deletions. Nucleic Acids Res. 2018; 46:e123. 30085201 10.1093/nar/gky691 PMC6237738

[17] Triche T.J. Jr, Weisenberger D.J., Van Den Berg D., Laird P.W., Siegmund K.D Low-level processing of Illumina infinium DNA methylation BeadArrays. Nucleic. Acids. Res. 2013; 41:e90. 23476028 10.1093/nar/gkt090 PMC3627582

[18] Leek J.T., Johnson W.E., Parker H.S., Jaffe A.E., Storey J.D. The sva package for removing batch effects and other unwanted variation in high-throughput experiments. Bioinformatics. 2012; 28:882–883. 22257669 10.1093/bioinformatics/bts034 PMC3307112

[19] Colaprico A., Silva T.C., Olsen C., Garofano L., Cava C., Garolini D., Sabedot T.S., Malta T.M., Pagnotta S.M., Castiglioni I. et al. TCGAbiolinks: an R/Bioconductor package for integrative analysis of TCGA data. Nucleic Acids Res. 2016; 44:e71. 26704973 10.1093/nar/gkv1507 PMC4856967

[20] VanderWeele T.J., Vansteelandt S. Odds ratios for mediation analysis for a dichotomous outcome. Am. J. Epidemiol. 2010; 172:1339–1348. 21036955 10.1093/aje/kwq332 PMC2998205

[21] Jo B., Stuart E.A., MacKinnon D.P., Vinokur A.D. The use of propensity scores in mediation analysis. Multivariate Behavioral Research. 2011; 46:425–452. 22399826 10.1080/00273171.2011.576624 PMC3293166

[22] Naimi A.I., Moodie E.E.M., Auger N., Kaufman J.S. Constructing inverse probability weights for continuous exposures: a comparison of methods. Epidemiology. 2014; 25:292–299. 24487212 10.1097/EDE.0000000000000053

[23] Mo Q., Wang S., Seshan V.E., Olshen A.B., Schultz N., Sander C., Powers R.S., Ladanyi M., Shen R. Pattern discovery and cancer gene identification in integrated cancer genomic data. Proc. Natl. Acad. Sci. U.S.A. 2013; 110:4245–4250. 23431203 10.1073/pnas.1208949110 PMC3600490

[24] Tian S., Wang C. An ensemble of the iCluster method to analyze longitudinal lncRNA expression data for psoriasis patients. Hum. Genomics. 2021; 15:23. 33879268 10.1186/s40246-021-00323-6 PMC8056592

[25] Wang T., Shao W., Huang Z., Tang H., Zhang J., Ding Z., Huang K. MOGONET integrates multi-omics data using graph convolutional networks allowing patient classification and biomarker identification. Nat. Commun. 2021; 12:3445. 34103512 10.1038/s41467-021-23774-w PMC8187432

[26] Kuleshov M.V., Jones M.R., Rouillard A.D., Fernandez N.F., Duan Q., Wang Z., Koplev S., Jenkins S.L., Jagodnik K.M., Lachmann A. et al. Enrichr: a comprehensive gene set enrichment analysis web server 2016 update. Nucleic. Acids. Res. 2016; 44:W90–W97. 27141961 10.1093/nar/gkw377 PMC4987924

[27] Dougan M., Dranoff G., Dougan S.K. GM-CSF, IL-3, and IL-5 family of cytokines: regulators of inflammation. Immunity. 2019; 50:796–811. 30995500 10.1016/j.immuni.2019.03.022 PMC12512237

[28] Chang S.H., Dong C. IL-17F: regulation, signaling and function in inflammation. Cytokine. 2009; 46:7–11. 19233684 10.1016/j.cyto.2008.12.024 PMC2663007

[29] Bernucci L., Henríquez M., Díaz P., Riquelme G. Diverse calcium channel types are present in the human placental syncytiotrophoblast basal membrane. Placenta. 2006; 27:1082–1095. 16564089 10.1016/j.placenta.2005.12.007

[30] Zhao Y., Pasanen M., Rysä J. Placental ion channels: potential target of chemical exposure. Biol. Reprod. 2022; 108:41–51. 10.1093/biolre/ioac186 PMC9843680 36173899

[31] Lee G., Kil G., Kwon J., Kim S., Yoo J., Shin J. Oncostatin M as a target biological molecule of preeclampsia. J. Obstet. Gynaecol. Res. 2009; 35:869–875. 20149034 10.1111/j.1447-0756.2009.01114.x

[32] Smith D.D., Costantine M.M. The role of statins in the prevention of preeclampsia. Am. J. Obstet. Gynecol. 2022; 226:S1171–S1181. 32818477 10.1016/j.ajog.2020.08.040 PMC8237152

[33] Liu X., Yin Z., Xu L., Liu H., Jiang L., Liu S., Sun X. Upregulation of LINC01426 promotes the progression and stemness in lung adenocarcinoma by enhancing the level of SHH protein to activate the hedgehog pathway. Cell Death. Dis. 2021; 12:173. 33568633 10.1038/s41419-021-03435-y PMC7875967

[34] Giroux-Leprieur E., Costantini A., Ding V.W., He B. Hedgehog signaling in lung cancer: from oncogenesis to cancer treatment resistance. Int. J. Mol. Sci. 2018; 19:2835. 30235830 10.3390/ijms19092835 PMC6165231

[35] Bon E., Driffort V., Gradek F., Martinez-Caceres C., Anchelin M., Pelegrin P., Cayuela M.-L., Marionneau-Lambot S., Oullier T., Guibon R. et al. SCN4B acts as a metastasis-suppressor gene preventing hyperactivation of cell migration in breast cancer. Nat. Commun. 2016; 7:13648. 27917859 10.1038/ncomms13648 PMC5150224

[36] Zhang C., Guo H., Li B., Sui C., Zhang Y., Xia X., Qin Y., Ye L., Xie F.a., Wang H. et al. Effects of Slit3 silencing on the invasive ability of lung carcinoma A549 cells. Oncol. Rep. 2015; 34:952–960. 26045181 10.3892/or.2015.4031

[37] Yang C., Yuan H., Gu J., Xu D., Wang M., Qiao J., Yang X., Zhang J., Yao M., Gu J. et al. ABCA8-mediated efflux of taurocholic acid contributes to gemcitabine insensitivity in human pancreatic cancer via the S1PR2-ERK pathway. Cell Death Discov. 2021; 7:6. 33431858 10.1038/s41420-020-00390-z PMC7801517

[38] Moh M.C., Shen S. The roles of cell adhesion molecules in tumor suppression and cell migration. Cell Adh. Migr. 2009; 3:334–336. 19949308 10.4161/cam.3.4.9246 PMC2802741

[39] Ferrando L., Vingiani A., Garuti A., Vernieri C., Belfiore A., Agnelli L., Dagrada G., Ivanoiu D., Bonizzi G., Munzone E. et al. ESR1 gene amplification and MAP3K mutations are selected during adjuvant endocrine therapies in relapsing Hormone Receptor-positive, HER2-negative breast cancer (HR+ HER2- BC). PLoS Genet. 2023; 19:e1010563. 36595552 10.1371/journal.pgen.1010563 PMC9839248

[40] De Santo I., McCartney A., Migliaccio I., Di Leo A., Malorni L. The emerging role of ESR1 mutations in luminal breast cancer as a prognostic and predictive biomarker of response to endocrine therapy. Cancers. 2019; 11:1894. 31795152 10.3390/cancers11121894 PMC6966519

[41] Acun T., Incekara O. High DNAJA4 expression correlates with poor survival outcomes in breast cancer. Rev. Romana Med. Laborator. 2022; 30:369–378.

[42] Paplomata E., O’Regan R The PI3K/AKT/mTOR pathway in breast cancer: targets, trials and biomarkers. Ther. Adv. Med. Oncol. 2014; 6:154–166. 25057302 10.1177/1758834014530023 PMC4107712

[43] Stemke-Hale K., Gonzalez-Angulo A.M., Lluch A., Neve R.M., Kuo W.-L., Davies M., Carey M., Hu Z., Guan Y., Sahin A. et al. An integrative genomic and proteomic analysis of PIK3CA, PTEN, and AKT mutations in breast cancer. Cancer Res. 2008; 68:6084–6091. 18676830 10.1158/0008-5472.CAN-07-6854 PMC2680495

[44] Peng Y., Wang Y., Zhou C., Mei W., Zeng C. PI3K/Akt/mTOR pathway and its role in cancer therapeutics: are we making headway?. Front. Oncol. 2022; 12:819128. 35402264 10.3389/fonc.2022.819128 PMC8987494

[45] Martínez-Sáez O., Chic N., Pascual T., Adamo B., Vidal M., González-Farré B., Sanfeliu E., Schettini F., Conte B., Brasó-Maristany F. et al. Frequency and spectrum of PIK3CA somatic mutations in breast cancer. Breast Cancer Res. 2020; 22:45. 32404150 10.1186/s13058-020-01284-9 PMC7222307

[46] Kazanietz M.G., Caloca M.J. The Rac GTPase in cancer: from old concepts to new paradigms. Cancer Res. 2017; 77:5445–5451. 28807941 10.1158/0008-5472.CAN-17-1456 PMC5645227

[47] Ayob A.Z., Ramasamy T.S. Cancer stem cells as key drivers of tumour progression. J. Biomed. Sci. 2018; 25:20. 29506506 10.1186/s12929-018-0426-4 PMC5838954

