# Supplementary material for: CWGCNA: an R package to perform causal inference from the WGCNA framework
Source: NAR Genom Bioinform. 2024 Apr 25;6(2):lqae042. doi: 10.1093/nargab/lqae042 (PMC11044439; doi:10.1093/nargab/lqae042)
Supplement: lqae042_Supplemental_Files [file lqae042_supplemental_files.zip › cwgcna_supplementarydata.pdf]

Supplementary Data

Contents

Supplementary Figures **2**

Supplementary Methods **11**

Data collection and preprocessing (*imputemeta* and *probestogenes*) 11

Causal *WGCNA* analysis (*diffwgcna*) 12

Multi-omics clustering (*multiCCA* and *wgcnacluster*) 16

Multi-omics classification (*omicsclassifier* and *pairedensemblepredict*) 18

Network-based gene set function annotation (*diffwgcna*) 21

Comparison with other public tools (*iClusterPlus*, *MOGONET*, and *EnrichR*) 23

References **25**

Supplementary Figures

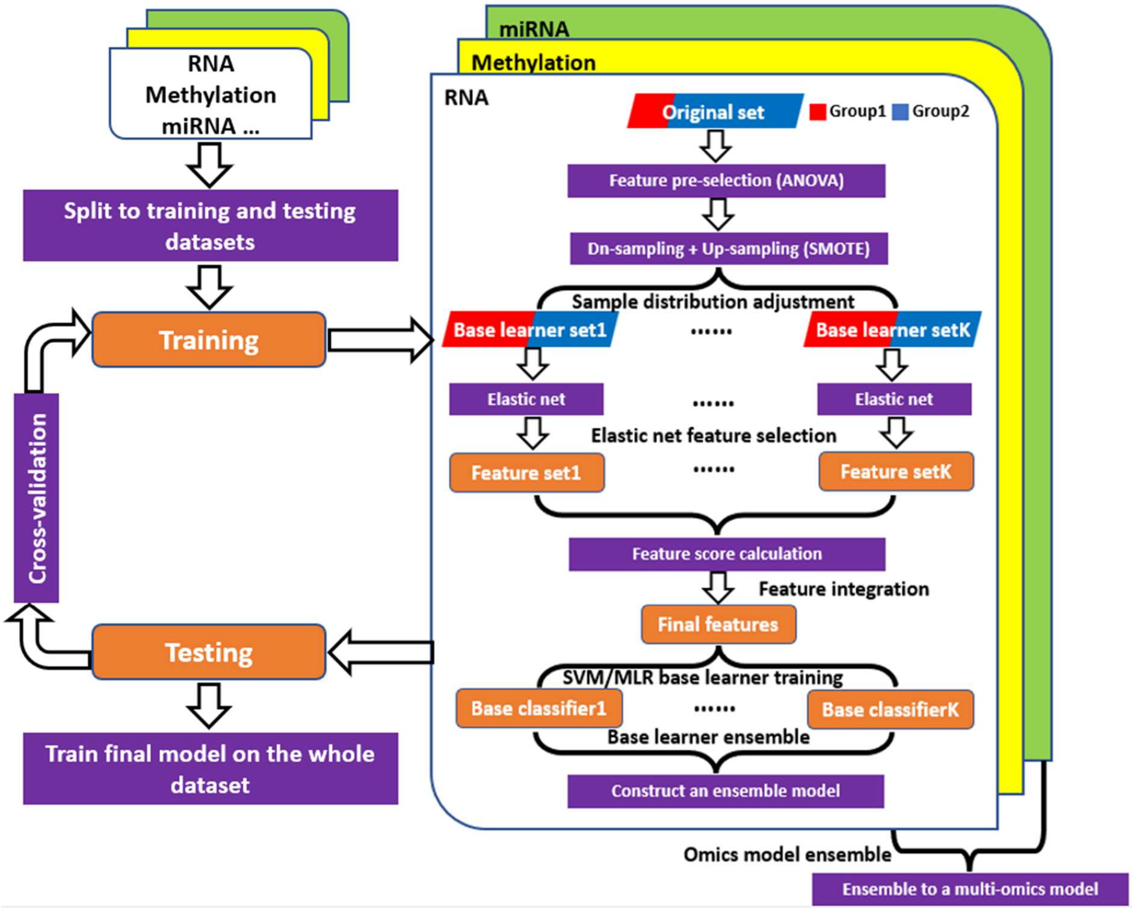

Figure S1. Workflow of the bagging-SMOTE model. The function *omicsclassifier* constructs a bagging-SMOTE model with several steps: sample distribution adjustment (bagging coupled with SMOTE), elastic net base learner feature selection, selected feature integration, SVM or MLR base leaner training, base learner ensemble, and omics model ensemble.

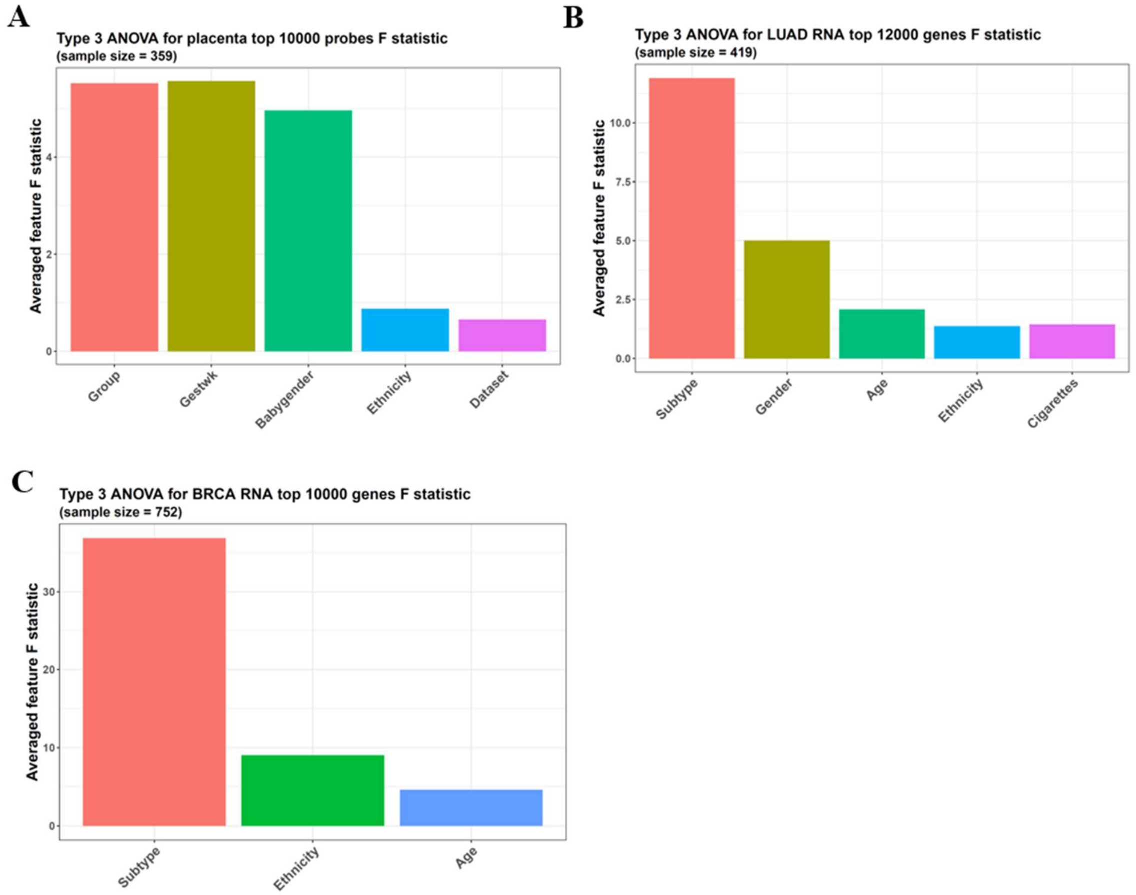

Figure S2. The ANOVA results of the function *featuresampling*. (A) For each phenotypic variable shown by the x-axis, *featuresampling* calculates its averaged F statistic across the top 10000 most variable DNAm features in the placenta dataset. Because this dataset is collected from various public ones, the variance of batch differences is also checked, and the F statistic of the variable Dataset is < 1, validating the effect of batch correction on the data. (B) For the top 12000 most variable genes in the LUAD RNA dataset, all the phenotypic variables on the x-axis have an F statistic > 1. (C) For the top 10000 most variable genes in the BRCA RNA dataset, the BRCA subtype group, ethnicity, and patient age show an F statistic > 1.

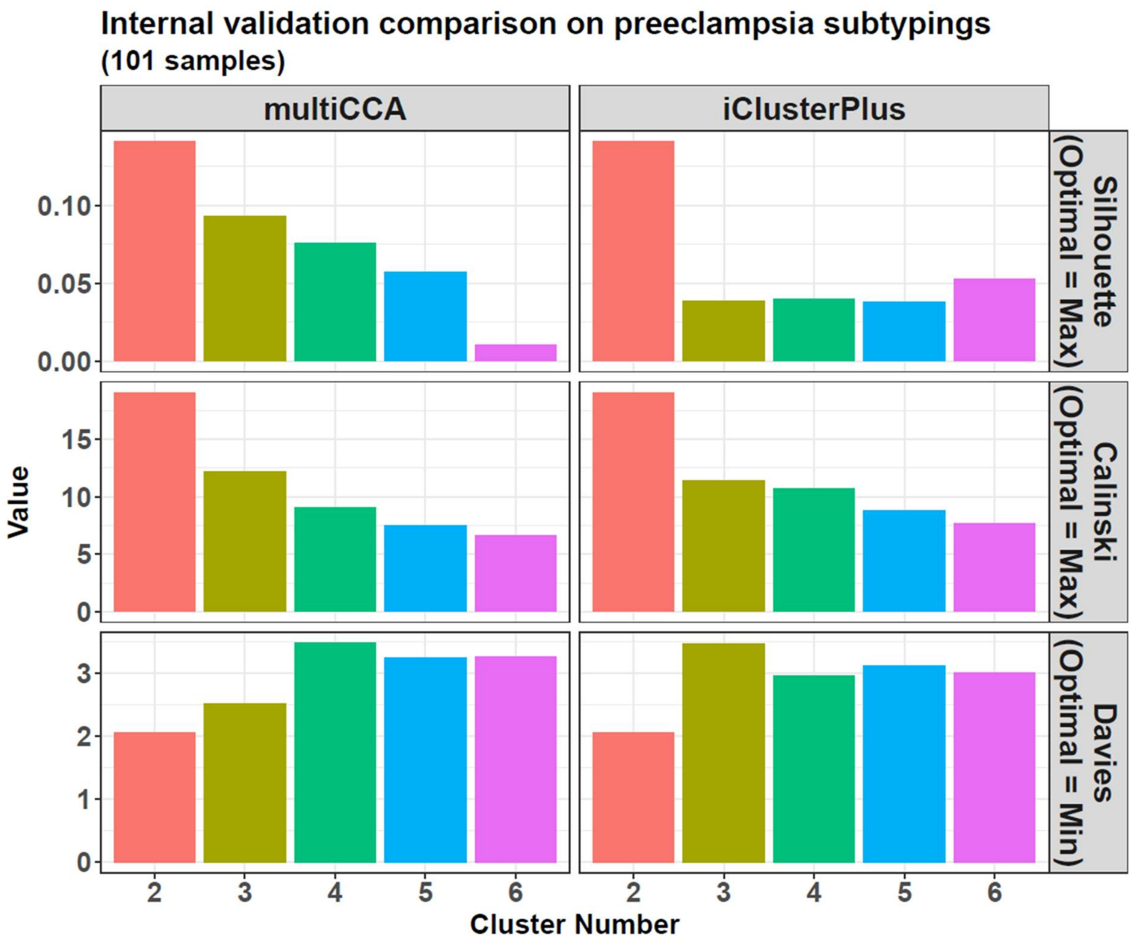

Figure S3. Internal validation indices for the preeclampsia DNAm data clustering. The *multiCCA* and *iClusterPlus* methods give the same clustering result when the sample cluster number is 2, with the Silhouette index = 0.141, the Calinski index = 19, and the Davies index = 2.05. These indices are calculated from the DNAm data based on the clustering result. When the cluster number changes from 3 to 6, the Silhouette and Calinski indices become smaller, and the Davies index becomes larger, meaning these results are weaker than the 2-cluster one.

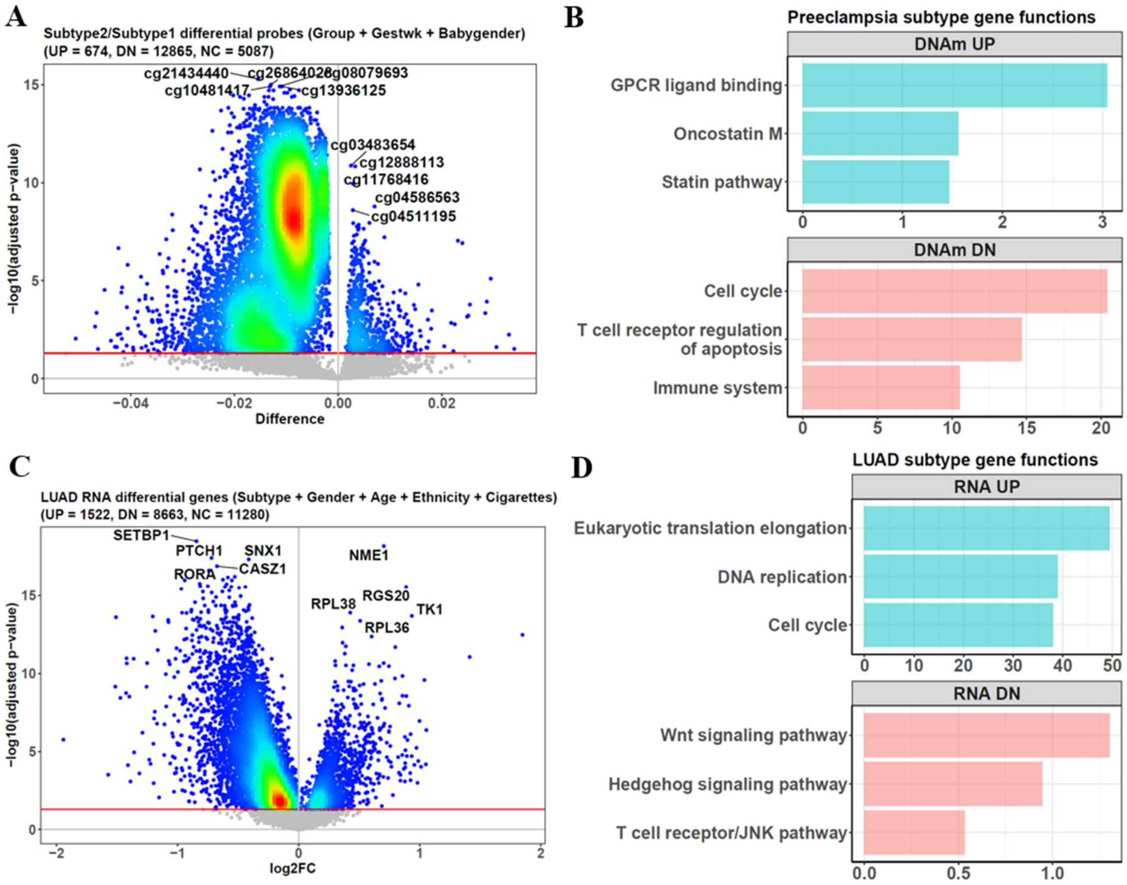

Figure S4. Inter-subtype comparisons for the preeclampsia and LUAD data. (A) Differential feature identification analysis shows that 674 DNAm probes are hyper-methylated in subtype2 preeclampsia samples relative to subtype1, and 12865 probes are hypo-methylated. The confounding factors are gestational week and baby gender, which have been adjusted during the analysis. The probes with an adjusted p-value < 0.05 are represented as colorful dots. (B) Functional enrichment for the subtype2/subtype1 differential DNAm features. The x-axis is -log10(adjusted p-value). (C) Differential feature identification analysis shows that 1522 genes are up-regulated in subtype2 LUAD samples relative to subtype1, and 8663 genes are down-regulated. The confounding factors are patients' gender, age, ethnicity, and cigarettes used per day. They have been adjusted during the analysis. The genes with an adjusted p-value

< 0.05 are represented as colorful dots. (D) Functional enrichment for the LUAD subtype2/subtype1 differential RNA genes. The x-axis is -log10(adjusted p-value).

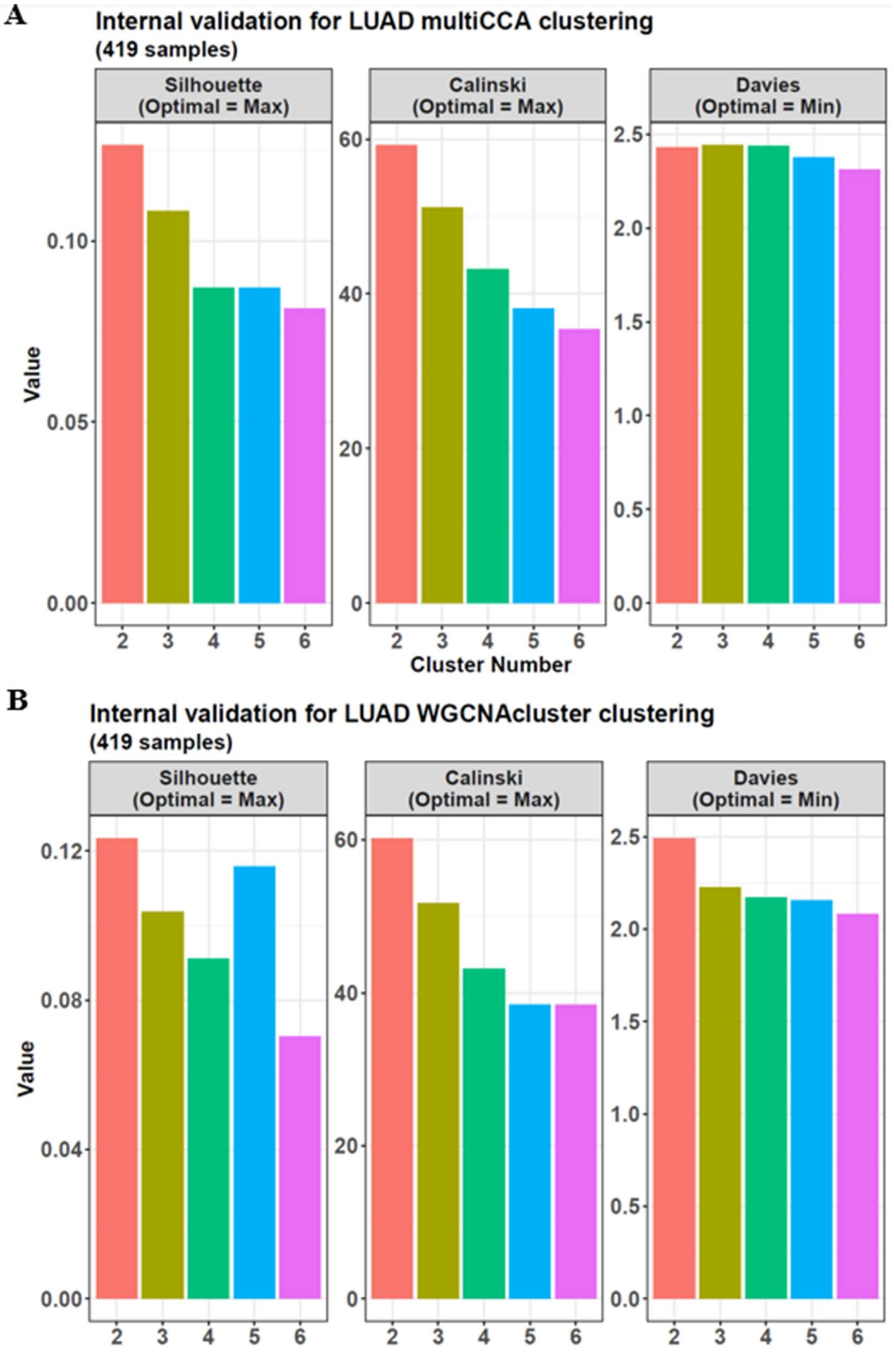

Figure S5. Internal validation indices for the LUAD multi-omics data clustering. (A) The *multiCCA* method gets optimal clustering when the sample cluster number is 2, with the

Silhouette index = 0.126, the Calinski index = 59.2, and the Davies index = 2.43. These indices are calculated from the integrated data generated by *multiCCA*. When the cluster number changes from 3 to 6, the Silhouette and Calinski indices become smaller, indicating weaker performance. (B) The *wgcnacluster* method also gets optimal clustering when the sample cluster number is 2, with its Silhouette index = 0.123, Calinski index = 60.2, and Davies index = 2.49. They are calculated from the integrated data generated by *wgcnacluster*. When the cluster number changes from 3 to 6, the Silhouette and Calinski indices decrease.

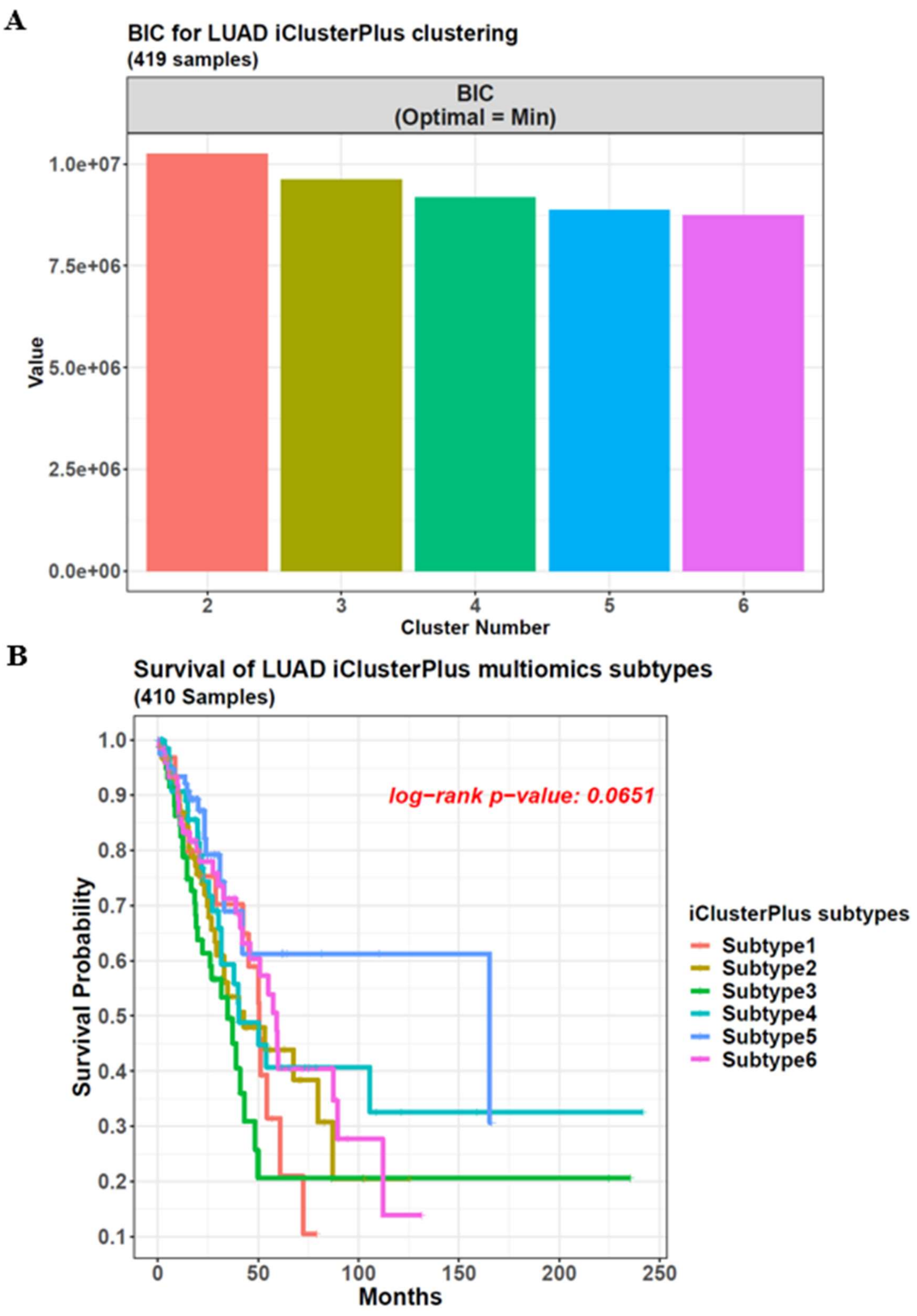

Figure S6. The *iClusterPlus* method clusters the LUAD multi-omics data into 6 sample groups. (A) When the cluster number is 6, *iClusterPlus*'s internally calculated BIC value

reaches its minimum (BIC = 8739347), meaning this result is optimal. (B) The survival difference among these 6 sample clusters from the LUAD multi-omics data. Because only 410 samples of the original 419 ones have survival information, only they are included here.

Supplementary Methods

Data collection and preprocessing (*imputemeta* and *probestogenes*)

The Infinium 27K and 450K data on control and preeclampsia human placentas were obtained from 10 GEO datasets: GSE31781 (1), GSE36829, GSE59274 (2), GSE74738 (3), GSE69502 (4), GSE98224 (5,6), GSE125605, GSE100197, GSE75196, and GSE73375. Then, we used *SeSAMe* to perform data preprocessing and merged the datasets so that only the overlapping probes shared by the Illumina 27K and 450K datasets were kept (7,8). The batch difference was adjusted via *ComBat* with GSE98224 data as the reference (9).

The above 10 datasets contained 359 placenta samples (258 control and 101 preeclampsia) and provided the gestational weeks for all of them (from 8 wk to 44.6 wk). In addition, 210 of the 359 samples had baby gender information, and 102 of the 359 samples had ethnicity information. To impute the missing values for baby gender and ethnicity, we used the gender prediction and ethnicity prediction models provided by *SeSAMe*, which predicted them with the methylation beta values. For baby gender, all its missing values could be predicted. Hence, the baby gender information became complete after the prediction, with 210 samples with an original label and 149 with a predicted label. For ethnicity, 257 samples were unlabeled, and 179 could be predicted from their methylation beta values.

Then, the missing ethnicities of the other 78 samples were imputed via the function *imputemeta* in our package. It was based on the *MICE* (multivariate imputations by chained equations) algorithm, which did not impute from beta values but from other phenotypic variables using Gibbs sampling (10). After that, the ethnicity information was also complete. Hence, the placenta dataset covered 359 samples. In addition to the methylation data, it contained their

phenotypic data on 4 variables: preeclampsia/control group, gestational week, baby gender, and ethnicity.

The RNA, methylation, and miRNA LUAD data were obtained from TCGA, with 419 cancer samples. The RNA and miRNA omics were downloaded as read count tables and converted to log2(TPM + 1). The DNA methylation (DNAm) data were 450K beta values, and the missing values were imputed with the k-nearest neighbors (KNN) method. Some specific DNAm probes in the data were filtered out, including non-CpG probes, multi-hint probes, SNP probes, and sex chromosome probes. The clinical data were also from TCGA, with missing values imputed by *imputemeta*.

The 752 BRCA cancer samples were from TCGA, and their BRCA PAM50 subtype information was from the R package *TCGAbiolinks* (11). Their RNA-seq read counts, 450K DNAm beta values, miRNA-seq read counts, and clinical data were downloaded. Then, the RNA and miRNA data were converted to log2(TPM + 1) values. For the DNAm probe data, after being preprocessed similarly to the LUAD one, they were converted to gene data with our function *probestogenes*, which averaged the probe beta values in gene TSS200, TSS1500, and 1stExon regions as the corresponding gene beta values.

Causal *WGCNA* analysis (*diffwgcna*)

The function *diffwgcna* performed causal *WGCNA* analysis. First, it used the traditional *WGCNA* method to calculate *WGCNA* modules from the data. Then, it compared the module eigengenes between sample groups by *limma*, identifying the modules related to the group difference. Using *limma* to find the differential eigengenes avoided the confounding factors' influence because *limma* regression removed their variance, so it was better than the traditional

WGCNA pipeline linking the modules and the phenotype using a correlation coefficient. Furthermore, within each module, *limma* could also be used on its nodes (features) to find the features related to the sample group difference.

Both the *limma* regression and the traditional correlation methods intended to connect the modules and features with the sample group difference. However, this connection was undirected, ignoring the potential causal relationships among the sample groups, module features, and modules. Hence, *diffwgcna* used mediation models to find any directed connections, which could be triggered by setting its parameter *mediation* as TRUE. The mediation models were the core of causal *WGCNA* and were constructed with the product method (12-14).

Let Y denote an outcome, A an exposure of interest, M a potential mediator, and C a set of baseline covariates, and the corresponding lower-case letters represent realizations of these random variables.

In the case that Y and M were continuous, *diffwgcna* would construct a linear model using the exposure A, the mediator M, and the covariates C to fit the outcome Y as $E[Y|a,m,c] = \theta_0 + \theta_1 a + \theta_2 m + \theta_3' c$, in which the coefficient θ_1 was interpreted as the direct effect of A on Y (NDE, natural direct effect). Also, a second model would be constructed to regress the mediator on the exposure and the covariates as $E[M|a,c] = \beta_0 + \beta_1 a + \beta_2' c$. Then, the product of β_1 and θ_2 was considered as the indirect effect of A on Y (NIE, natural indirect effect), which was the effect of the exposure on the mediator times that of the mediator on the outcome. Hence, $NDE = \theta_1$ and $NIE = \theta_2\beta_1$.

On the other hand, if M were continuous but Y were binary, a logistic regression model

would be constructed for the outcome as $logit[P(Y = 1|a, m, c)] = \theta_0 + \theta_1 a + \theta_2 m + \theta_3' c$, and the mediator model would still be the linear one as $E[M|a, c] = \beta_0 + \beta_1 a + \beta_2' c$. Then, NDE and NIE would still be $NDE = \theta_1$ and $NIE = \theta_2 \beta_1$, but they were on an odds ratio scale.

After that, in the assumption that M mediated the effect of A on Y, the proportion mediated (PCT) was calculated with NDE and NIE. In detail, if NDE and NIE had the same sign, then $PCT = NIE/(NDE + NIE)$. However, if NDE and NIE had opposite signs, which meant there was a suppression mediation effect, then $PCT = |NIE/NDE|$. The 95% confidential intervals (CIs) of NDE, NIE, and PCT were estimated via bootstrapping 100 times. Accordingly, if the upper and lower confidence limits of NIE had the same sign, a mediation effect would be considered significant. Furthermore, if the upper and lower confidence limits of NDE also had the same sign, but NDE and NIE had opposite signs, the mediation would be annotated as a suppression effect.

In addition, IPW (inverse probability weighting) was used to inflate the weights for under-represented observations. If exposure A were binary, covariates C would be used to predict it with logistic regression, and the fitted values for the samples would be the propensity scores to calculate inverse probability weights. A sample with the exposure $A = a$ would get a weight to be used in the mediator model as $\frac{a}{propensity} + \frac{1-a}{1-propensit}$ (15).

On the other hand, if A were continuous, its probability distribution would be estimated as a Gaussian one of $f_A(A; \mu_1, \sigma_1^2)$, where μ_1 and σ_1^2 were A's mean and variance across the dataset. At the same time, a linear model would be used to predict A with covariates C, generating the probability distribution for each sample as $f_{A|C}(A|C = c; \mu_2, \sigma_2^2)$, where μ_2

and σ_2^2 were the predictions and residual variance of the model. Then, for a sample with $A = a$ and $C = c$, its weight would be $\frac{f_A(A=a;\mu_1,\sigma_1^2)}{f_{A|C}(A=a|C=c;\mu_2,\sigma_2^2)}$ (16).

The function *diffwgcna* constructed the mediation models after identifying the inter-group differential features within each module. For RNA expression data, a module meant a group of genes whose transcription profiles were highly correlated, and the features were the genes in that group (17). A module's eigengene and a gene's expression level were used when constructing a mediation model, and for each feature in a specific module, *diffwgcna* built 2 models. The first one tested the mediation relationship of "module→module feature→group", which meant the module caused the sample group difference using its feature as the mediator. The second tested the opposite direction of "group→module feature→module", which meant the sample group difference caused the module feature to change, and finally, the module also changed. Hence, both used the feature as the potential mediator M (continuous variable). The difference was that the first one used the feature's module as the exposure A (continuous module eigengene values) and used the sample group as the outcome Y (binary variable), but the second one swapped them. Finally, the significant features mediating their module's effect on the group, or *vice versa*, were identified by the 95% CIs of the 2 models. Sometimes, a feature was significant in both models and would be discarded.

If *diffwgcna*'s parameter *balanceadj* were FALSE, the above mediation analysis would be performed on the modules of the original dataset.

However, if this parameter were TRUE, it would generate several base learner datasets from the original one. Then, it performed the mediation tests on each base learner's modules and ensembled their results to get the final. The base learners were derived from the original data

via a bagging-SMOTE (synthetic minority over-sampling technique) framework. It adjusted the data distribution via the bagging and SMOTE sampling methods, so in this case, any sample size difference between the original sample groups would be balanced. It could prevent the group size difference from impairing the test power. This step was implemented similarly to another function, *omicsclassifier*, in the package, which utilized it to enhance the sample label prediction for rare samples.

In addition, *diffwgcna* needed to ensure that the base learners' modules covered the same features, i.e., the features in different base learners had the same module assignments so that their results could be ensembled. Hence, *diffwgcna* shared the module assignments from the original dataset with all the base learners.

After that, the mediation tests were performed on each of them, and ensemble was used to get the final result. In this step, for the mediation NDE, it would be the mean of all the base leaner $NDEs$. Its upper confidence limit would be $\overline{NDE} + \sqrt{\frac{\sum_{i=1}^{n}(\widehat{NDE_i} - NDE_i)^2}{n}}$, where $\overline{NDE}$ was the mean of all the base learner $NDEs$, NDE_i was the NDE of the ith base learner, and $\widehat{NDE_i}$ was its upper confidence limit. The lower confidence limit would be calculated similarly, and the NIE and CPT results would also be aggregated in this way.

Multi-omics clustering (*multiCCA* and *wgcnacluster*)

The function *multiCCA* performed multi-omics clustering. It first scaled the omics data, so all the features had a unified mean of 0 and a standard deviation of 1. If only 2 omics were used for clustering, the covariance matrix between them would be calculated, and CCA (canonical correlation analysis) would be used on it, generating the top 30 CC components for each omic. Hence, in each single-omic dataset, all the samples got 30 new features (the CCs), and a matrix

was formed. Its row number was the sample number, and its column number was the CC number. In total, 2 such matrices were generated for the 2 omics. At the same time, a weight was calculated for each matrix, determined by the variance it explained for its single-omic data. Next, the weights of the 2 matrices were scaled to the sum of 1, and their weighted sum was calculated, generating a merged matrix. If a third omic existed, this merged matrix would be treated as a single omic, and the third omic was the other. The same process was used to merge them, and if there were also the fourth or other omics, this process would be repeated to combine all of them into one merged matrix, which would undergo k-means clustering.

To determine the optimal k value for the k-means clustering, *multiCCA* accepted multiple candidate k values. Then, for each of them, 3 internal validation indices were calculated after the clustering. They were: 1) the Silhouette index as $Silhouette = \frac{1}{k}\sum_{i=1}^{k}\left\{\frac{1}{|C_i|}\sum_{x \in C_i}\frac{b(x)-a(x)}{\max\,[b(x),a(x)]}\right\}$, where $a(x) = \frac{1}{|C_i|-1}\sum_{y \in C_i, x \neq y} d(x,y)$, $b(x) = min_{j,j \neq i}\left[\frac{1}{|C_j|}\sum_{y \in C_j} d(x,y)\right]$, 2) the Calinski index as $Calinski = \frac{\sum_i^k |C_i| d^2(c_i,c)/(n-1)}{\sum_i^k \sum_{x \in C_i} d^2(x,c_i)/(n-k)}$, and 3) the Davies-Bouldin index as $Davis - Bouldin = \frac{1}{k}\sum_i^k max_{j,j \neq i}\left\{\left[\frac{1}{|C_i|}\sum_{x \in C_i} d(x,c_i) + \frac{1}{|C_j|}\sum_{x \in C_j} d(x,c_j)\right]/d(c_i,c_j)\right\}$, where $d(x,y)$ was the distance between samples x and y on the merged matrix, C_i was the ith cluster, c_i was its center, c was the center of the whole data. The optimal k value was the one with the largest Silhouette and Calinski indices and the smallest Davis-Bouldin index.

If the dataset was not multi-omics but single-omic, *multiCCA* could also perform the above process to cluster. However, the CCA would be changed to PCA on the covariance matrix of the single-omic dataset.

In addition, our package contained another function, *wgcnacluster*, which could also perform

multi-omics clustering and had almost the same steps as *multiCCA*. However, its method of generating the merged matrix before k-means clustering differed. For each omic, *wgcnacluster* did not use CCA but used *WGCNA* to generate the *WGCNA* module eigengenes for its samples. Then, the ones from different omics were directly combined, generating a final matrix containing multi-omics *WGCNA* module eigengenes. After that, *k*-means would be used on it to perform the clustering.

Multi-omics classification (*omicsclassifier* and *pairedensemblepredict*)

In addition to clustering, our package could also perform multi-omics classification, depending on the function *omicsclassifier*. It scaled the omics data first, giving all the features a unified mean of 0 and a standard deviation of 1 across the samples. Then, it performed type-III ANOVA on each feature to screen the ones with a p-value < 0.05 to the response variable (sample classes), and they would be used for the classification following several steps (Figure S1).

For each single-omic dataset, if the parameter *balanceadj* were set as 1, *omicsclassifier* would adjust the data distribution and generate 10 balanced base learner datasets. In this case, the original big and small sample classes would finally have the same sample numbers. This was achieved by conducting up or down-sampling on each class until its final sample number reached the ceiling of $\frac{dataset\ sample\ number}{class\ number}$. Hence, if a class contained too many samples, it would be down-sampled, but if a class only had a few samples, it would be up-sampled.

To implement the up-sampling, *omicsclassifier* used SMOTE to synthesize new samples via interpolation. If a sample were randomly selected, 1 of its 5 nearest neighbors in the same class would also be sampled. Then, a vector recording the feature value difference between this neighbor and the selected sample would be calculated. Meanwhile, a random number between

0 and 1 would be generated to multiply this vector, and the new vector would be added to the original sample. The result was the synthesized sample with the same class label.

On the other hand, because the large classes lost some samples during down-sampling, a bagging framework was introduced to rescue these samples, so an ensemble model was constructed on the whole dataset. It contained 10 base learner datasets with balanced sample classes. If a base learner did not select a sample during down-sampling, it could still be selected by other base learners, so all the samples could be used for the whole ensemble. This bagging-SMOTE framework was also used by the function d*iffwgcna* to perform the causal *WGCNA* analysis in an ensemble manner.

After getting the 10 balanced base learner datasets, *omicsclassifier* used each of them to train a base learner via elastic net regularization, $\min_{(\beta_{0k},\beta_k)\in\mathbb{R}^{p+1}} \left[\frac{1}{N}\sum_{i=1}^{N}(-\sum_{k=1}^{K} y_{il}\,(\beta_{0k} + x_i^T\beta_k) + \log\,(\sum_{l=1}^{K} e^{\beta_{0l}+x_i^T\beta_l}))\right] + \lambda\left[(1-\alpha)\,\frac{1}{2}\,\parallel \beta \parallel_F^2 + \alpha\sum_{j=1}^{p} \parallel \beta_j \parallel_1\right]$ (18,19). In the formula, the first part was the multinomial negative log-likelihood, and the second was the multiclass elastic net penalty. The α parameter was set as 0.5, which controlled the balance of L1 and L2 penalties. At the same time, the regularization constant λ was chosen during a 10-fold cross-validation. The function chose the α-λ combination giving the minimum cross-validation error and used it to construct the elastic net model.

Each base learner used this elastic net method to select a set of features. Then, their features were combined, and those with the top scores were selected, which were calculated referring to a previous method predicting drug responses (20). It used the formula, $\left(\mathcal{F}_p^+ - \mathcal{F}_p^-\right) * \overline{\beta_p}$, where $\mathcal{F}_p^+ = \frac{1}{K}\sum_{k=1}^{K} I\left(\beta_p^{(k)} > 0\right)$ and $\mathcal{F}_p^- = \frac{1}{K}\sum_{k=1}^{K} I\left(\beta_p^{(k)} < 0\right)$ represented the percentages of base learners with a coefficient larger or smaller than 0 for the *p*th feature selected for a

specific sample class, and $\overline{\beta_p} = \frac{1}{K}\sum_{k=1}^{K}\beta_p^{(k)}$ was the averaged coefficient value of this feature across all the K base learners.

Then, the selected features were returned to each base learner dataset to generate an SVM (support vector machine) or MLR (multinomial logistic regression) model. Finally, these new base learners were ensembled together, which was achieved by assigning each of them a weight as $0.5 * \log\frac{ACC}{1-ACC}$, where ACC was the base learner prediction accuracy on the original training dataset. The predicted class distribution of the whole ensemble was the weighted sum of the base learner ones. Only the base learners with an ACC > 0.5 would be included in the ensemble, and the base learner weights would be scaled to the sum of 1.

If the parameter *balanceadj* were set as 1, the above bagging-SMOTE sampling could balance the sample distribution for the downstream model training. However, if *balanceadj* were set as 2, *omicsclassifier* would not perform this bagging-SMOTE sampling or adjust data distribution. Instead, it would train a normal bagging model using bootstrapping to generate base learner datasets. Hence, bootstrapping models could be built in this manner. The parameter *balanceadj* could also be set as 3. In this case, neither the bagging-SMOTE sampling nor the bootstrapping would be conducted, so *omicsclassifier* would skip the base learner generation step and directly use the original training data to perform the elastic net feature selection and the SVM/MLR model training.

In addition, if the data transferred to *omicsclassifier* were single-omic, the above process would return the final classification result. However, if the data were multi-omics, the process would be used on each single-omic first. Then, their predicted class distributions would be further aggregated with the weighted sum method above. This time, the weights were not calculated

from each base learner but from each single-omic model accuracy.

Moreover, *omicsclassifier* also contained a parameter *nfold*, which could assign a fold number to perform cross-validation training. If it were NULL, this step would be skipped, and only the classifier trained from the whole data would be returned. It could be further transferred to another function, *pairedensemblepredict*, which predicted new sample classes with this classifier.

Network-based gene set function annotation (*diffwgcna*)

The *WGCNA* modules' gene set function annotation could be performed by *diffwgcna*. Unlike traditional enrichment methods that only covered a module's nodes (genes), *diffwgcna* further considered its network structure, reflected by the network edges and their weights.

In our edge-based analysis, the function terms of each edge (gene-gene pair) were defined as the intersection of its 2 genes'. Then, 2 methods could be used on them to perform gene set function annotation: 1) edge weight shuffling and 2) hypergeometric test.

For the edge weight shuffling method, when it was applied to a *WGCNA* module, all the edges there exchanged their weight values mutually and randomly. Hence, each of them would get a new weight value originally belonging to another edge.

Notably, only their weights were shuffled, and their function terms were not. Then, *diffwgcna* focused on the function terms. For each of them, all the edges with this term would be found, and their weights would be summed up and used as this function's weight. Because the edges never exchanged their function terms, after a shuffling, the same edges would be found as those with a specific function. However, these edges' weights were changed during the shuffling, so their weight sum would change, i.e., the function's weight would change after each shuffling.

Such shuffling would be performed 1000 times, so a function would get 1000 different weights, forming a weight distribution. Furthermore, its original weight calculated from the original module edge weights would be mapped to this distribution to get a p-value, indicating whether this function's original weight was significantly large.

For the other hypergeometric test method, if not considering weights, the test for a function term could be calculated with 4 numbers, including 1) N: the total number of edges in the background, 2) n: that in the module, 3) M: the number of edges with that function term in the background, and 4) m: that in the module. Hence, the final p-value was $P(X > k) = \sum_{m=k+1}^{\min(M,n)} \frac{\binom{M}{m}\binom{N-M}{n-m}}{\binom{N}{n}}$, where k was the realization of m in the module.

If further considering edge weights in the enrichment, the hypergeometric test would become difficult because it needed to calculate combinations, such as $\binom{N}{n} = \frac{N!}{(N-n)!n!}$, where the factorials required non-negative integers. However, the edge weights were not restricted to integers. Hence, *diffwgcna* referred to a gene-based enrichment tool, *WEAT*, which solved this problem by introducing the Γ function (21). Similarly, *diffwgcna* used it because it was an extension of factorial to complex numbers as $x! = \Gamma(x+1) = \int_0^\infty t^x e^{-t} dt$, where x was a complex number. Accordingly, the hypergeometric test on edge weights became $P(X > k) = \int_k^{\min(M,n)} \frac{\binom{M}{m}\binom{N-M}{n-m}}{\binom{N}{n}} dm$. The factorials for calculating $\binom{N}{n}$ were solved by the Γ function, and N and n were the total edge weights in the background and the module. In addition, the edge weights were normalized in advance as $w_i' - \frac{1}{n}\sum_{i=1}^{n} w_i' + 1$, where $w_i' = \frac{w_i - \min(W)}{\max(W) - \min(W)}$, and W was the vector of all edge weights, including the ith as w_i.

However, because *WGCNA* constructed a fully-connected network across all the input genes, the gene pair number in the background would be huge due to the gene combination, bringing

a burden of calculating so many gene pairs. Hence, only the top 1% edges with the highest TOM (topological overlap matrix) weights would be considered as the background, and for a module, only its edges belonging to this top 1% would be in the enrichment analysis.

For both the shuffling and hypergeometric methods, their p-values would be adjusted with Benjamini-Hochberg correction.

Comparison with other public tools (*iClusterPlus, MOGONET,* and *EnrichR*)

For multi-omics or single-omic clustering, the performance of *CWGCNA* was compared with the public tool *iClusterPlus*, which was the enhanced version of *iCluster*, a widely used method to integrate multiple genomics data and cluster the samples using a joint latent variable model (22,23). If the dataset contained RNA or miRNA omic, their log2(TPM + 1) values were used. If the dataset had DNAm omic, the probe M values were transferred to the function *iClusterPlus* in the R package *iClusterPlus*. In addition, the function's parameter *K* was set from 1 to 5, meaning to cluster the samples into 2 to 6 clusters. Then, its internally calculated BIC values were used to compare the results of different cluster numbers and select the optimal one. For other parameters, they were set as their default values.

For multi-omics classification, *CWGCNA* was compared with the *MOGONET* model. It was a graph convolutional network (GCN) specially developed for multi-omics classification and was reported as better than other methods (24). Before starting the *MOGONET* training, we conducted feature selection following its original study. Briefly, for the DNAm data, only probes contained in the Infinium 27K platform were retained, and the ones with a variance < 0.001 or mean = 0 across the samples were filtered out. For the mRNA-seq data, the genes with a variance < 0.1 or mean = 0 were removed. For the miRNA-seq data, the miRNA genes with

a variance = 0 or mean = 0 were removed. After that, ANOVA was used on each feature across different sample classes, and only the ones with an adjusted p-value < 0.05 were kept for the *MOGONET* model construction. In addition, these filtered data's PC1 (first principal component) should explain < 50% of the data variance. Finally, because *MOGONET* required that the features in each omic should be within [0, 1], a scaling step was conducted on the data before the model training.

For *WGCNA* module gene set function annotation, *EnrichR* was used for gene-node-based enrichment (25). The function *enrichr* in the R package *enrichR* accepted the gene names via its parameter *genes*, and the other parameter *databases* was used to specify the background databases for the enrichment analysis. In this study, the GO and Reactome ones were chosen. The returned result gave several parameters, including *Adjusted.P.value* and *P.value*, and if a function term had an *Adjusted.P.value* < 0.05, its enrichment was deemed significant. However, if < 10 terms in the results reached this criterion, *P.value* < 0.01 would be used as a relatively loose criterion to determine the significant function terms.

The edge-based method in *CWGCNA* used the same *Adjusted.P.value* or *P.value* to identify the significant functions. When using its intersection strategy to get the edge function terms, it was also based on the GO and Reactome databases.

References

1. Novakovic, B., Yuen, R.K., Gordon, L., Penaherrera, M.S., Sharkey, A., Moffett, A., Craig, J.M., Robinson, W.P. and Saffery, R. (2011) Evidence for widespread changes in promoter methylation profile in human placenta in response to increasing gestational age and environmental/stochastic factors. *BMC Genomics*, **12**, 529.
2. Chu, T., Bunce, K., Shaw, P., Shridhar, V., Althouse, A., Hubel, C. and Peters, D. (2014) Comprehensive analysis of preeclampsia-associated DNA methylation in the placenta. *PLoS One*, **9**, e107318.
3. Hanna, C.W., Peñaherrera, M.S., Saadeh, H., Andrews, S., McFadden, D.E., Kelsey, G. and Robinson, W.P. (2016) Pervasive polymorphic imprinted methylation in the human placenta. *Genome research*, **26**, 756-767.
4. Price, E.M., Peñaherrera, M.S., Portales-Casamar, E., Pavlidis, P., Van Allen, M.I., McFadden, D.E. and Robinson, W.P. (2016) Profiling placental and fetal DNA methylation in human neural tube defects. *Epigenetics Chromatin*, **9**, 6.
5. Leavey, K., Wilson, S.L., Bainbridge, S.A., Robinson, W.P. and Cox, B.J. (2018) Epigenetic regulation of placental gene expression in transcriptional subtypes of preeclampsia. *Clin Epigenetics*, **10**, 28.
6. Wilson, S.L., Leavey, K., Cox, B.J. and Robinson, W.P. (2018) Mining DNA methylation alterations towards a classification of placental pathologies. *Human molecular genetics*, **27**, 135-146.
7. Zhou, W., Triche, T.J., Jr., Laird, P.W. and Shen, H. (2018) SeSAMe: reducing artifactual detection of DNA methylation by Infinium BeadChips in genomic deletions. *Nucleic Acids Res*, **46**, e123.
8. Triche, T.J., Jr., Weisenberger, D.J., Van Den Berg, D., Laird, P.W. and Siegmund, K.D. (2013) Low-level processing of Illumina Infinium DNA Methylation BeadArrays. *Nucleic Acids Res*, **41**, e90.
9. Leek, J.T., Johnson, W.E., Parker, H.S., Jaffe, A.E. and Storey, J.D. (2012) The sva package for removing batch effects and other unwanted variation in high-throughput experiments. *Bioinformatics*, **28**, 882-883.
10. van Buuren, S. and Groothuis-Oudshoorn, K. (2011) mice: Multivariate Imputation by Chained Equations in R. *Journal of Statistical Software*, **45**, 1 - 67.
11. Colaprico, A., Silva, T.C., Olsen, C., Garofano, L., Cava, C., Garolini, D., Sabedot, T.S., Malta, T.M., Pagnotta, S.M., Castiglioni, I. *et al.* (2016) TCGAbiolinks: an R/Bioconductor package for integrative analysis of TCGA data. *Nucleic Acids Res*, **44**, e71.
12. Ferguson, K.K., Chen, Y.-H., VanderWeele, T.J., McElrath, T.F., Meeker, J.D. and Mukherjee, B. (2017) Mediation of the Relationship between Maternal Phthalate Exposure and Preterm Birth by Oxidative Stress with Repeated Measurements across Pregnancy. *Environmental Health Perspectives*, **125**, 488-494.
13. VanderWeele, T.J. (2016) Mediation Analysis: A Practitioner's Guide. *Annual Review of Public Health*, **37**, 17-32.
14. VanderWeele, T.J. and Vansteelandt, S. (2010) Odds Ratios for Mediation Analysis for a Dichotomous Outcome. *American Journal of Epidemiology*, **172**, 1339-1348.
15. Jo, B., Stuart, E.A., MacKinnon, D.P. and Vinokur, A.D. (2011) The Use of Propensity Scores in Mediation Analysis. *Multivariate Behavioral Research*, **46**, 425-452.
16. Naimi, A.I., Moodie, E.E.M., Auger, N. and Kaufman, J.S. (2014) Constructing Inverse Probability Weights for Continuous Exposures: A Comparison of Methods. *Epidemiology*, **25**, 292-299.
17. Zhang, B. and Horvath, S. (2005) A General Framework for Weighted Gene Co-Expression Network

Analysis. *Statistical Applications in Genetics and Molecular Biology*, **4**.
18. Friedman, J., Hastie, T. and Tibshirani, R. (2010) Regularization Paths for Generalized Linear Models via Coordinate Descent. *Journal of statistical software*, **33**, 1-22.
19. Tibshirani, R., Bien, J., Friedman, J., Hastie, T., Simon, N., Taylor, J. and Tibshirani, R.J. (2012) Strong rules for discarding predictors in lasso-type problems. *Journal of the Royal Statistical Society. Series B, Statistical methodology*, **74**, 245-266.
20. Ding, Z., Zu, S. and Gu, J. (2016) Evaluating the molecule-based prediction of clinical drug responses in cancer. *Bioinformatics*, **32**, 2891-2895.
21. Fan, R. and Cui, Q. (2021) Toward comprehensive functional analysis of gene lists weighted by gene essentiality scores. *Bioinformatics*, **37**, 4399-4404.
22. Mo, Q., Wang, S., Seshan, V.E., Olshen, A.B., Schultz, N., Sander, C., Powers, R.S., Ladanyi, M. and Shen, R. (2013) Pattern discovery and cancer gene identification in integrated cancer genomic data. *Proceedings of the National Academy of Sciences*, **110**, 4245-4250.
23. Tian, S. and Wang, C. (2021) An ensemble of the iCluster method to analyze longitudinal lncRNA expression data for psoriasis patients. *Human Genomics*, **15**, 23.
24. Wang, T., Shao, W., Huang, Z., Tang, H., Zhang, J., Ding, Z. and Huang, K. (2021) MOGONET integrates multi-omics data using graph convolutional networks allowing patient classification and biomarker identification. *Nature Communications*, **12**, 3445.
25. Kuleshov, M.V., Jones, M.R., Rouillard, A.D., Fernandez, N.F., Duan, Q., Wang, Z., Koplev, S., Jenkins, S.L., Jagodnik, K.M., Lachmann, A. *et al.* (2016) Enrichr: a comprehensive gene set enrichment analysis web server 2016 update. *Nucleic acids research*, **44**, W90-97.
