# Supplementary material for: CWGCNA: an R package to perform causal inference from the WGCNA framework
Source: NAR Genom Bioinform. 2024 Apr 25;6(2):lqae042. doi: 10.1093/nargab/lqae042 (PMC11044439; doi:10.1093/nargab/lqae042)
Supplement: lqae042_Supplemental_Files [file lqae042_supplemental_files.zip › tutorial_CWGCNA.docx]

Tutorial for R package CWGCNA

Yu Liu

2023-11-10

## Introduction

*WGCNA* (weighted gene co-expression network analysis) is a very useful tool for identifying co-expressed gene modules and detecting their correlations to phenotypic traits *[1]*. Here, we explored more possibilities about it and developed the R package *CWGCNA* (causal *WGCNA*), which works from the traditional *WGCNA* pipeline but mines more information. It couples a mediation model with *WGCNA*, so the causal relationships among *WGCNA* modules, module features, and phenotypes can be found, demonstrating whether the module change causes the phenotype change or *vice versa*. After that, when annotating the module gene set functions, it uses a novel network-based method, considering the modules’ topological structures and capturing their influence on the gene set functions. In addition to conducting these biological explorations, *CWGCNA* also contains a machine learning section to perform clustering and classification on multi-omics data, given the increasing popularity of this data type. Some basic functions, such as differential feature identification, are also available in our package. This tutorial will introduce the main functions of *CWGCNA*.

## Package installation

The code of *CWGCNA* is freely available at <https://github.com/yuabrahamliu/CWGCNA>.

The following commands can be used to install this R package, and an R version >= 4.2.3 is required.

library(devtools)

install_github("yuabrahamliu/CWGCNA")

## Data preparation

This tutorial will use the data accompanying the *CWGCNA* package. They contain a matrix covering DNA methylation (DNAm) data from 359 human placenta samples, which are collected from 10 different GEO datasets based on Illumina 27K and 450K platforms. The matrix columns represent the samples, and the rows represent DNAm probes. Totally 18626 probes are in the matrix and are shared high-quality probes among the samples. The probe values are beta values and have been preprocessed with batch effect adjusted. Among the 359 samples, 258 are normal samples, and the remaining 101 are disease samples with preeclampsia pregnancy complications caused by placental abnormalities.

In addition to this matrix, a data frame with this package records the metadata for the 359 samples. It includes the sample IDs (column “sampleid”, corresponding to the column names of the beta value matrix), their preeclampsia/control status (column “Group”), the gestational ages (weeks) of the samples (column “Gestwk”), the baby gender (column “Babygender”), the maternal ethnicity (column “Ethnicity”), and the original GEO dataset IDs of the samples (column “Dataset”).

Attach *CWGCNA* to the R session and look at these data.

library(CWGCNA)

betas <- system.file("extdata", "placentabetas.rds", package = "CWGCNA")
betas <- readRDS(betas)

pds <- system.file("extdata", "placentapds.rds", package = "CWGCNA")
pds <- readRDS(pds)

The beginning parts of these data are shown below.

#The DNAm betas matrix

betas[1:6,1:6]
#> GSM788417 GSM788419 GSM788420 GSM788421 GSM788414 GSM788415
#> cg00000292 0.65961366 0.67591141 0.65709651 0.66077820 0.66847653 0.67436406
#> cg00002426 0.53516824 0.53883284 0.53683120 0.53990206 0.53804637 0.53543344
#> cg00003994 0.17674229 0.16432771 0.16631494 0.16803355 0.16416337 0.16852233
#> cg00007981 0.03553198 0.02934814 0.03189098 0.02765783 0.02768899 0.02764183
#> cg00008493 0.43619912 0.42998023 0.43761396 0.44973257 0.46281094 0.45935308
#> cg00008713 0.07431056 0.06367040 0.06764089 0.06218142 0.05267028 0.05397539

#The metadata for the samples
head(pds)
#> sampleid Group Gestwk Babygender Ethnicity Dataset
#> 1 GSM788417 Control 8 M White GSE31781
#> 2 GSM788419 Control 8 M White GSE31781
#> 3 GSM788420 Control 8 M White GSE31781
#> 4 GSM788421 Control 9 M White GSE31781
#> 5 GSM788414 Control 12 F Asian GSE31781
#> 6 GSM788415 Control 12 M White GSE31781

table(pds$Group)
#>
#> Control Preeclampsia
#> 258 101

table(pds$Dataset)
#>
#> GSE100197 GSE125605 GSE31781 GSE36829 GSE59274 GSE69502 GSE73375 GSE74738
#> 65 41 30 48 23 16 36 28
#> GSE75196 GSE98224
#> 24 48

## Confounding factor analysis

We first check the relationship between the methylation beta values in the betas matrix and the phenotypic variables in the pds metadata using the function *featuresampling* in our package. It performs type-III ANOVA to measure the variance of each phenotypic variable explained for the beta value dataset.

We only perform the analysis on the top 10000 most variable DNAm probes in the data, which can be selected by *featuresampling*. Its parameter betas accepts our betas placenta data matrix and the parameter topfeatures can be set to 10000 so that the top 10000 most variable probes will be selected. Another parameter, variancetype, is used to define how to calculate the variance, and we set it to "sd", meaning that standard deviation will be used. The parameter threads defines the threads number for parallelization.

#This command will take < 1 min
top10k <- featuresampling(betas = betas,
 topfeatures = 10000,
 variancetype = "sd",
 threads = 6)

top10k$betas[1:6,1:6]
#> GSM788417 GSM788419 GSM788420 GSM788421 GSM788414 GSM788415
#> cg00000292 0.6596137 0.6759114 0.6570965 0.6607782 0.6684765 0.6743641
#> cg00002426 0.5351682 0.5388328 0.5368312 0.5399021 0.5380464 0.5354334
#> cg00003994 0.1767423 0.1643277 0.1663149 0.1680336 0.1641634 0.1685223
#> cg00008493 0.4361991 0.4299802 0.4376140 0.4497326 0.4628109 0.4593531
#> cg00013618 0.7859429 0.7868591 0.7933922 0.7958716 0.8006630 0.8118193
#> cg00014837 0.8522323 0.8525327 0.8516924 0.8546466 0.8610099 0.8546916

dim(top10k$betas)
#> [1] 10000 359

head(top10k$varicanceres)
#> SD
#> cg03729431 0.1848922
#> cg16063666 0.1643842
#> cg05790038 0.1456048
#> cg03316864 0.1454261
#> cg11009736 0.1393115
#> cg20322876 0.1348288

The result top10k is a list. Its slot betas contains the data matrix only with the top 10000 most variable DNAm probes, and the other slot varicanceres records the standard deviation of the original 18626 probes calculated by *featuresampling*. Then, we further use this function to perform ANOVA on the slot betas. We transfer our metadata pds to its parameter pddat, so all its variables except the first column “sampleid” will be analyzed by ANOVA. The parameter anova should be set as TRUE to make the function perform ANOVA.

The parameter plotannovares is used to indicate whether it is needed to plot the ANOVA result for the data, and featuretype and plottitilesuffix are characters that need to be shown in the plot title and can also be set as NULL. titilesize and textsize are used to control the font sizes of the figure title and texts.

#This command will take < 1 min
anovares <- featuresampling(betas = top10k$betas,
 pddat = pds,
 anova = TRUE,

 plotannovares = TRUE,
 featuretype = "probe",
 plottitlesuffix = "placenta",
 titlesize = 18,
 textsize = 16,

 threads = 6)


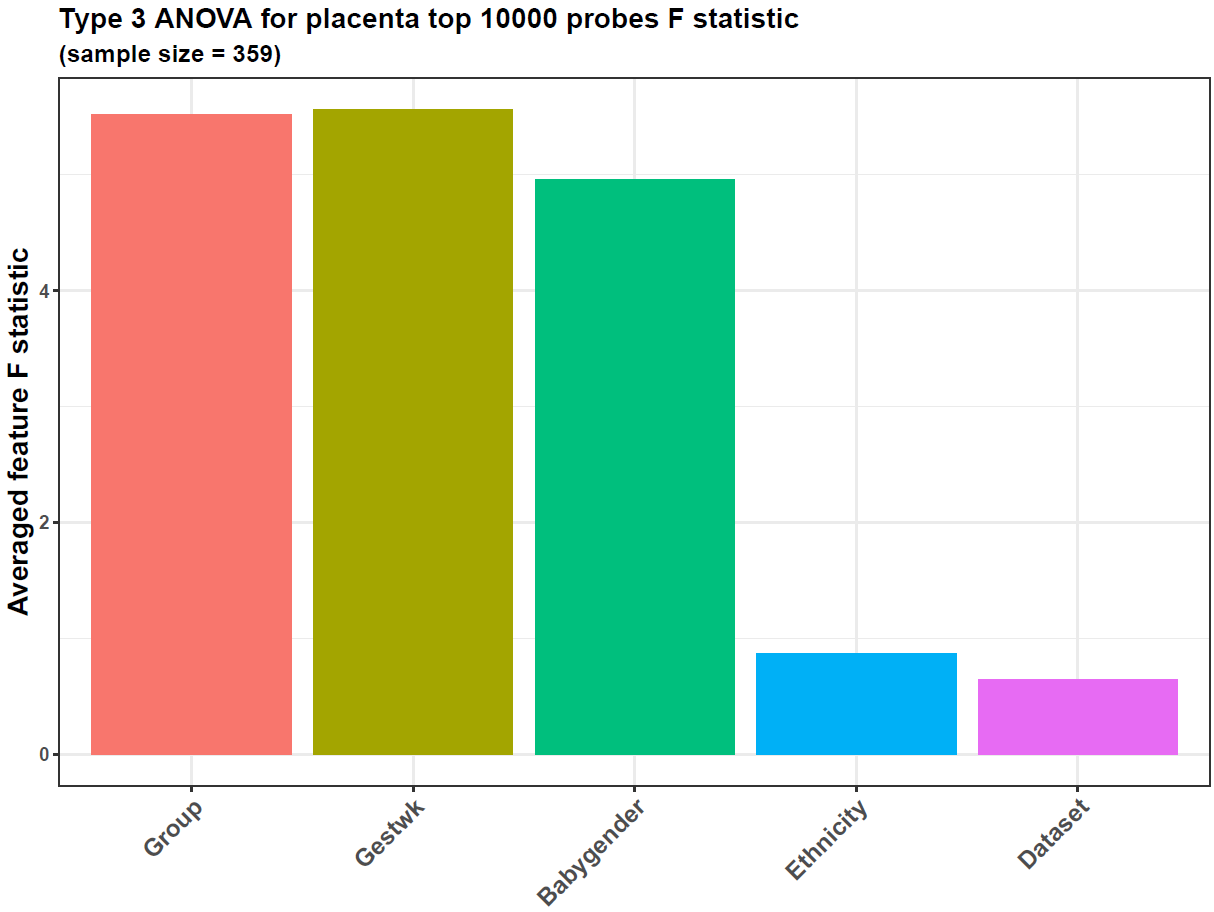


In this case, each probe in the data will go through ANOVA, and its result F statistic, MSS (mean sum of the square), and p-value will be contained in the slot varianceres in the list anovares returned by *featuresampling*. The plot generated shows the average F statistic across all the input probes.

From the plot, the preeclampsia/control sample group, gestational week, and baby gender account for the most variance, implying this dataset is suitable for checking the DNAm difference between the sample groups. However, the variances from gestational age and baby gender are also high, with an F value > 1. Hence, they are confounding factors for preeclampsia analysis, and their variance needs to be adjusted.

## Causal *WGCNA* analysis

Next, we conduct the causal *WGCNA* analysis on the data. However, before that, we use our function *probestogenes* to compress the DNAm probe beta values to genes.

#This command will take < 1 min
betasgene <- probestogenes(betadat = betas, group450k850k = c("TSS200", "TSS1500", "1stExon"))

The parameter group450k850k means that for a specific gene, the probe located in its TSS200, TSS1500, and 1stExon regions will be selected, and their mean value will be the gene’s beta value.

Then, we use another function, *diffwgcna*, to perform the causal *WGCNA* analysis. Its parameter dat accepts the betas matrix, and pddat accepts the pds data frame. The parameters responsevarname and confoundings are important because the former can define which column in pds is the target variable, and the latter can define which confoundings need to be adjusted, and we transfer "Group" to the former, and c("Gestwk", "Babygender") to the latter. All of them correspond to the column names in pds, and they are necessary for the causal *WGCNA* analysis. In addition, *diffwgcna* calls *limma* to find the significantly inter-group differential *WGCNA* modules and the differential features within each module. The parameters responsevarname and confoundings are also needed for the *limma* regression step called by *diffwgcna*.

Because we have converted the DNAm probe beta values to genes, we set the parameter featuretype as "gene". We also set the parameter topvaricancetype as "sd" and topvaricance as 5000, meaning we will perform this analysis on the top 5000 most variable genes in the data (the ones with the highest standard deviation).

The function, *diffwgcna*, performs its causal inference within the traditional *WGCNA* framework. Hence, it is necessary to call the traditional *WGCNA* modules. We transfer the vector seq(1, 20, 1) to powers, so grid search will be used to select the optimal soft-thresholding power from 1 to 20 for *WGCNA*. The desired minimum scale-free topology fitting index is 0.8 because we set another parameter, rsqcutline, as 0.8.

The parameter mediation is important because it controls whether mediation analysis, the core of causal inference, needs to be performed during the *WGCNA* pipeline. For each *WGCNA* module, it constructs mediation models to test 2 causal directions. One is “module->module gene->preeclampsia”, and the other is “preeclampsia->module gene->module”. Hence, we can determine whether the disease causes the module changes or the module drives the disease.

We set the parameter topn as 1, so the causal inference will be performed only on the top 1 most differential *WGCNA* module and its features. The default value of this parameter is NULL, and in this case, all the differential modules will go through the causal inference, but it is time-consuming to analyze all of them.

If the parameter plot were set to TRUE, several plots would be generated to show the results, and the characters transferred to titleprefix will be shown in the plot titles. The labelnum parameter indicates how many top features in the *limma* regression and causal inference results will be labeled in the plot. It can also be set to NULL so that no feature names will be marked. We set it to 5 here. The parameter annotextsize can control the font size of these feature labels.

The parameter pvalcolname is set as "adj.P.Val", and pvalcutoff is 0.05, indicating that the differential modules and genes identified by *limma* regression should have an adjusted p-value < 0.05. Because our data values here are DNAm beta values, we set the parameter isbetaval as TRUE. The parameter diffcutoff defines the cutoff on module eigengene difference to select differential modules, and the parameter absxcutoff defines the gene log2FC cutoff to select differential genes within the modules. Because the gene values are beta values here, the log2FC result of *limma* indicates the beta value difference between the groups.

This *WGCNA* analysis is time-consuming (~ 25 min), and the running below can be skipped.

#This command will take ~ 25 min
cwgcnares <- diffwgcna(dat = betasgene,
 pddat = pds,
 responsevarname = "Group",
 confoundings = c("Gestwk", "Babygender"),
 featuretype = "gene",

 topvaricancetype = "sd",
 topvaricance = 5000,

 powers = seq(1, 20, 1),
 rsqcutline = 0.8,

 mediation = TRUE,

 topn = 1,

 plot = TRUE,
 titleprefix = "Placenta",
 labelnum = 5,
 titlesize = 17,
 textsize = 16,
 annotextsize = 5,

 pvalcolname = "adj.P.Val",
 pvalcutoff = 0.05,
 isbetaval = TRUE,
 absxcutoff = 0,
 diffcutoff = 0,

 threads = 6)


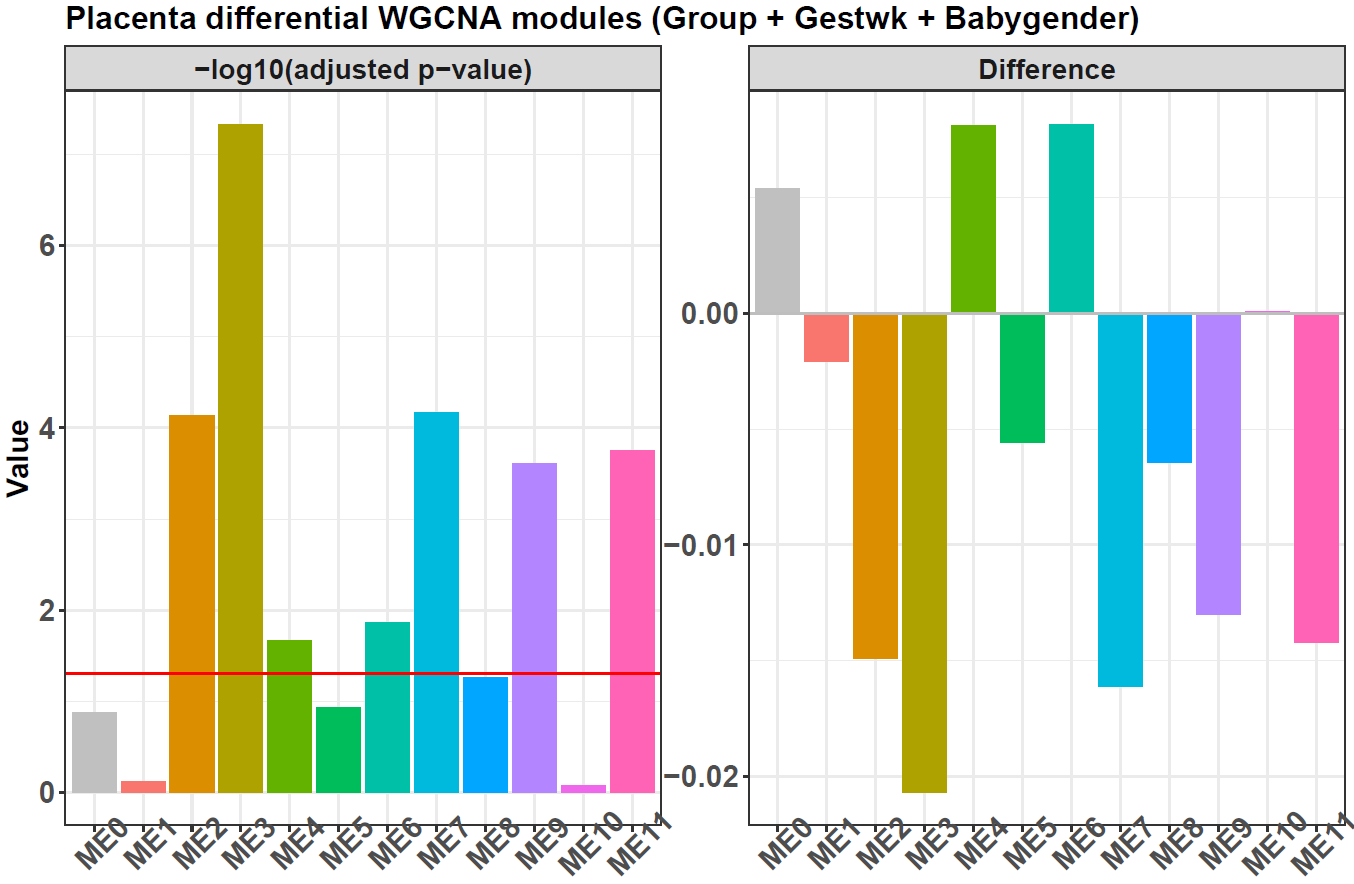


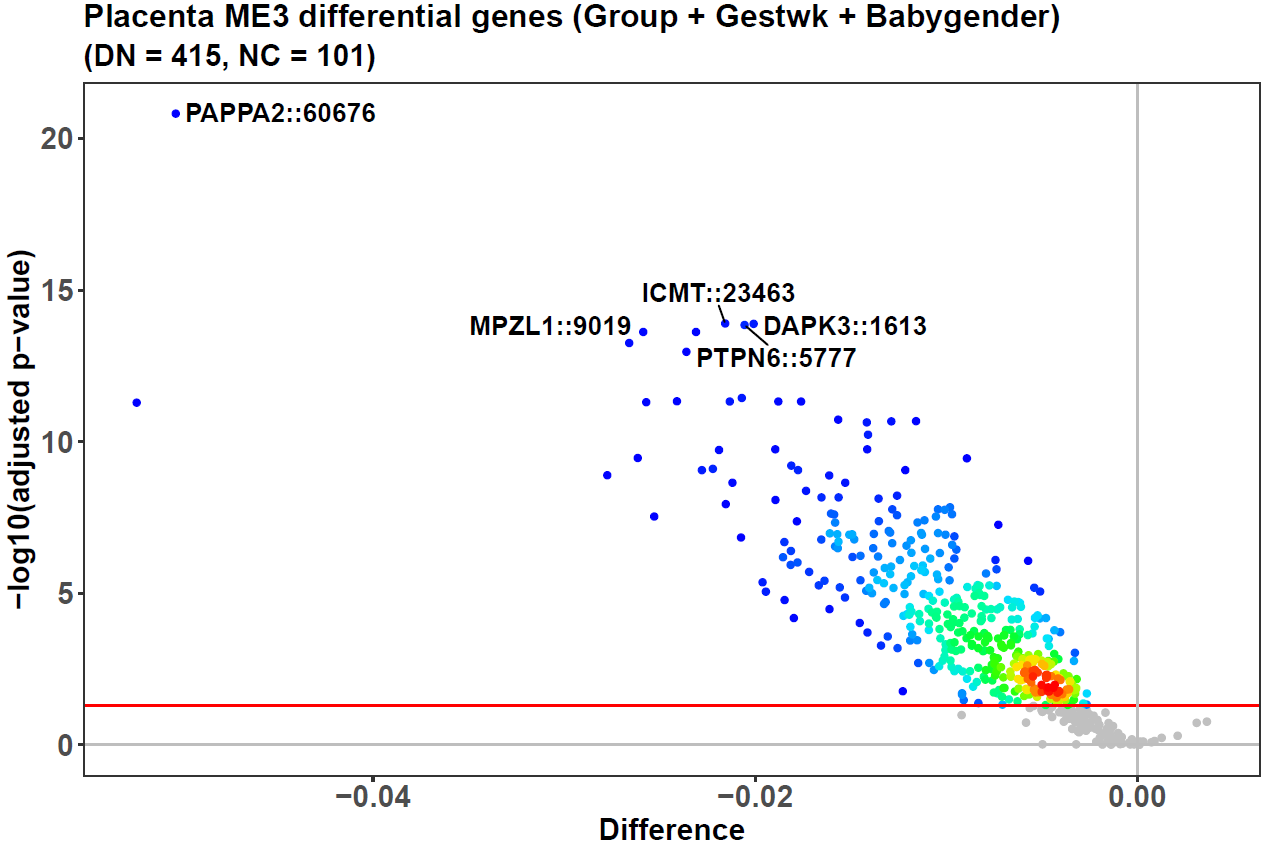


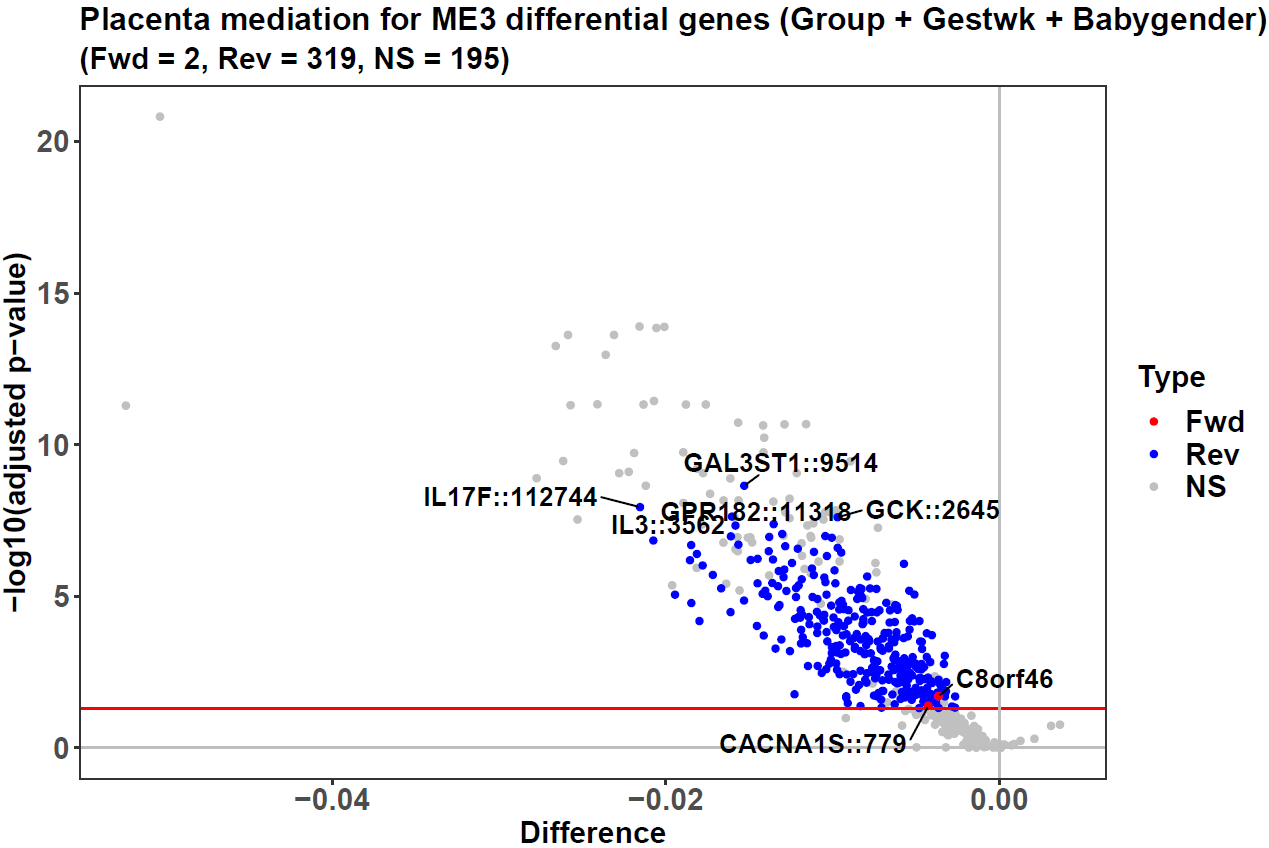


The result cwgcnares is a list containing several slots. The one named “limmares” is the *limma* result on all the *WGCNA* modules, and the bar plot generated by the function is based on this result. It shows the data contain 11 modules from ME1 to ME11, and 7 have significantly differential eigengenes between preeclampsia/control groups.

In addition, *diffwgcna* also calls *limma* to select the differential features within each module. For example, the volcano plot of ME3 shows 415 of its 516 genes are hypo-methylated in preeclampsia compared to the control. The slot “melimmares” contains the results within each significantly differential module.

Because the parameter mediation is set as TRUE, and the parameter topn is set as 1, the causal inference result for the most differential module, ME3, is returned, and the slot “mediationres” records it. Correspondingly, ME3 gets an additional volcano plot to show its mediation result. In this second volcano plot, the 319 blue dots are genes mediating the causal direction of “ME3->ME3 gene->preeclampsia” (the reverse direction), and the red dots mediate the direction of “preeclampsia->ME3 gene->ME3” (the forward direction). The y-axis and x-axis show the -log10(adjusted p-value) and preeclampsia/control beta value difference when screening the differential genes with *limma*.

The 319 genes are associated with inflammatory stress in preeclampsia, such as IL3 and IL17F, the top genes found in ME3. Both of them are cytokines for inflammation *[2,3]*. Because these genes’ causal direction is from preeclampsia to ME3, the conclusion is that inflammation is not the cause but the result of this disease.

Meanwhile, CACNA1S is the top gene mediating the causal direction from ME3 to preeclampsia. This gene has expression in the placenta *[4,5]*, and encodes a calcium channel responsible for vascular contraction. Given that preeclampsia is a pregnancy hypertension disease, the driving effect of CACNA1S on it is clear. Moreover, this gene is the target of amlodipine besylate, a small-molecule drug for preeclampsia treatment, further supporting that the inference here is reasonable.

## PCA/CCA-based clustering and bagging-SMOTE classification

Finally, we use our package to perform some machine-learning tasks. We first use the function *multiCCA* to cluster the 101 preeclampsia samples to see if any subtypes exist. The function *multiCCA* uses multiple CCA to convert multi-omics data into a compressed one and then performs k-means to get the clustering result. On the single-omic here, the multiple CCA becomes PCA for the DNAm data of the 101 samples.

#Extract the DNAm probe data for the 101 preeclampsia samples
prepds <- subset(pds, Group == "Preeclampsia")
row.names(prepds) <- 1:nrow(prepds)

prebetas <- betas[, prepds$sampleid, drop = FALSE]

#Clustering
#This command will take < 1 min
presubtyperes <- multiCCA(dats = list(prebetas),

 k = 2,
 consensus = 1,

 seednum = 2022,
 threads = 6,
 plot = TRUE,
 titlefix = "Preeclampsia",
 titlesize = 18,
 textsize = 16)


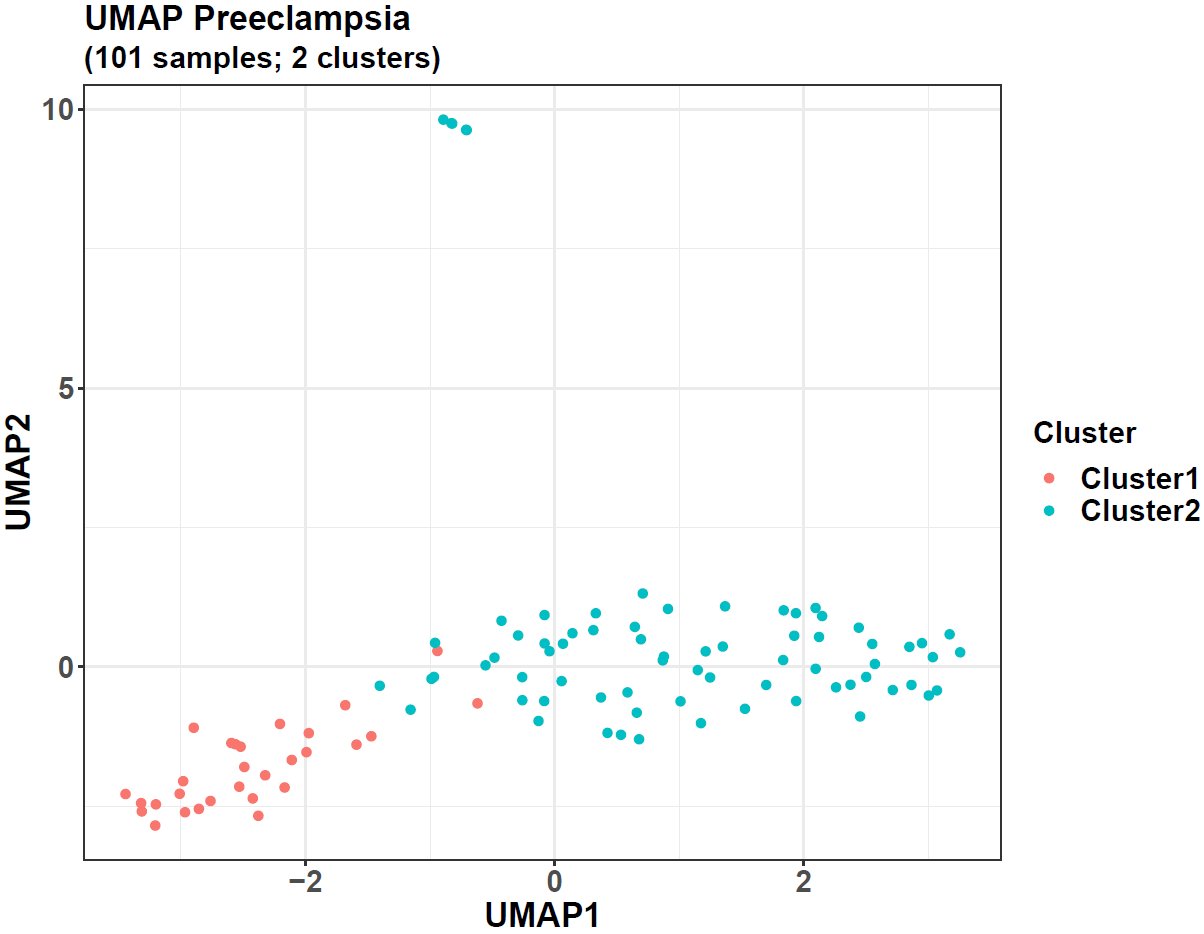


The parameter dats accepts a list with each element as the data of one omic. Because our current task is single-omic, we transfer a one-element list with the preeclampsia DNAm data.

The parameter k defines the cluster number for the k-means step, and if the optimal cluster number is not known, a vector, such as c(2, 3, 4), can be transferred, so for each number in it, the k-means step will use it as the cluster number. The optimal one can be defined by the internal validation indices calculated by *multiCCA*. From our previous experiment, the optimal cluster number for this preeclampsia dataset is 2, so we can directly use 2 here and do not need to transfer more candidate numbers. Another parameter, consensus, is used for consensus clustering. If we set it as 100, then consensus clustering will be conducted for each cluster number, and its feature shuffling will be performed 100 times. However, we set it as 1 here, and a normal clustering will be conducted without the consensus process.

Because the parameter plot is TRUE, the clustering results will be plotted.

The result presubtyperes is a list, and its slot “ivis” records 3 internal validation indices for each candidate cluster number.

presubtyperes$ivis
#> $`k = 2`
#> silhouette calinski_harabasz davies_bouldin
#> 0.2622399 36.6521374 1.3709824

We only transfer one number to the parameter k, so the Silhouette, Calinski, and Davies indices are only calculated for the cluster number of 2. If more candidate numbers are transferred, these indices will also be calculated for their clustering results, and the optimal cluster number should have larger Silhouette and Calinski indices but a smaller Davies index, which means that its intra-cluster samples are closer to each other and the inter-cluster sample distances are larger.

The slot “kreses” contains the cluster labels for samples, and its sub-slot “k = 2” is for the cluster number of 2.

head(presubtyperes$kreses$`k = 2`)
#> GSM1892055 GSM2674426 GSM2674432 GSM2589560 GSM2674433 GSM1892048
#> 2 1 2 2 2 1

table(presubtyperes$kreses$`k = 2`)
#>
#> 1 2
#> 29 72

Hence, 29 samples belong to Cluster1 (subtype1), and 72 belong to Cluster2 (subtype2). The function also returns their UMAP plot, and the 2 clusters are well separated, supporting the existence of 2 subtypes in the preeclampsia data.

After getting the subtype labels, we can train a classifier to classify the samples using the function *omicsclassifier*. It has an ensemble framework combining the bagging and the SMOTE (synthetic minority over-sampling technique) methods, which adjusts the imbalanced sample distribution so that the model can be trained from a balanced one. This makes the final model more accurate in rare sample label prediction, such as the small subtype1 class here.

In addition to balancing sample distribution, the bagging-SMOTE framework also performs feature selection with elastic net. Then, it uses these features to construct several SVM (support vector machine) or MLR (multinomial logistic regression) base learners and finally ensembles their results.

#Make the sample labels from the clustering result
subtypes <- paste0("Subtype", presubtyperes$kreses$`k = 2`)

#Classification
#This command will take < 5 min
presubtypeclassifierres <- omicsclassifier(dats = list(prebetas),
 truelabels = subtypes,

 balanceadj = 1,

 method = "SVM",

 alphas = c(0.5),
 nfold = 5,

 seednum = 2022,
 threads = 6,

 plot = TRUE,
 prefixes = c("Preeclampsia (SVM-balance)"))


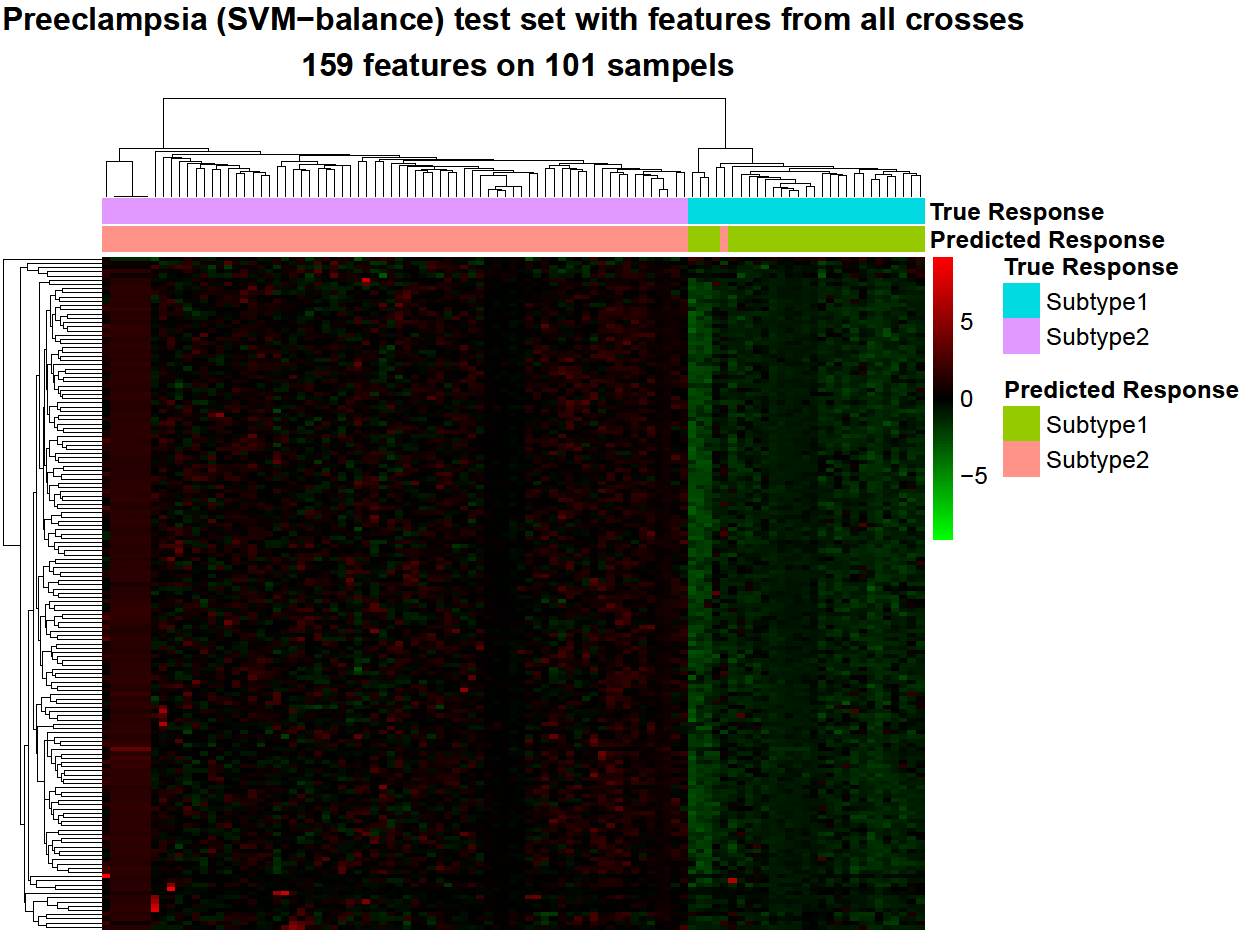


*omicsclassifier* can handle both single-omic and multi-omics data. In this case, we transfer the DNAm data of the preeclampsia samples to its parameter dats, similar to that in *multiCCA*. The parameter truelabels needs the subtype labels from the clustering result.

Then, balanceadj is set as 1, so it can trigger the bagging-SMOTE framework for model training. However, it can also be set as 2, and in this case, it will construct a normal bagging framework instead, which will not do any distribution adjustment. In addition, if it is set as 3, the bagging-SMOTE or normal bagging framework will be skipped, and only the feature selection and SVM/MLR model training steps will be conducted.

The parameter method is set as "SVM", so SVM base learners will be trained after the bagging-SMOTE and elastic net steps. Another parameter alphas accepts a number between 0 and 1. It controls the balance of L1 and L2 penalties in the elastic net feature selection. In addition, nfold indicates whether a cross-fold validation should be performed, and we set it as 5, so a 5-fold cross-validation will be used to evaluate the model’s performance. If it is set as NULL, cross-fold validation will not be performed, and only a model trained on the whole dataset will be returned.

The parameter plot is set as TRUE, so the function will generate some plots to show the final results.

The result presubtypeclassifierres contains a slot named “cvtestcomps”, showing the predicted and true labels for the testing data during the 5-fold cross-validation. The bagging-SMOTE framework for the model training will not influence the testing data’s sample distribution.

head(presubtypeclassifierres$cvtestcomps)
#> Prediction True
#> GSM1892031 Subtype2 Subtype2
#> GSM1892044 Subtype2 Subtype2
#> GSM1892047 Subtype1 Subtype1
#> GSM1892048 Subtype1 Subtype1
#> GSM1892055 Subtype2 Subtype2
#> GSM1892056 Subtype2 Subtype2

#Number of correct classifications
sum(as.character(presubtypeclassifierres$cvtestcomps$Prediction) == as.character(presubtypeclassifierres$cvtestcomps$True))
#> [1] 100

#Accuracy
sum(as.character(presubtypeclassifierres$cvtestcomps$Prediction) == as.character(presubtypeclassifierres$cvtestcomps$True))/nrow(presubtypeclassifierres$cvtestcomps)
#> [1] 0.990099

#Confusion matrix
table(presubtypeclassifierres$cvtestcomps)
#> True
#> Prediction Subtype2 Subtype1
#> Subtype2 72 1
#> Subtype1 0 28

#Accuracies for each sample class
diag(table(presubtypeclassifierres$cvtestcomps))/colSums(table(presubtypeclassifierres$cvtestcomps))
#> Subtype2 Subtype1
#> 1.0000000 0.9655172

From the slot “cvtestcomps”, we can see that 100 of the 101 samples are classified correctly, so the 5-fold cross-validation accuracy is 0.99. It is also shown by the heatmap returned by the function, where each column represents one sample, and each row represents one DNAm probe selected by the model. The entries are beta values after scaling across the samples.

Furthermore, if we check the model’s accuracies on each sample class, the misclassified sample is from the small class (a subtype1 sample), so it has an ACC of 0.966 on this small class.

On the other hand, if the parameter balanceadj is set as 2 or 3, the bagging model or the non-framework model will be trained, and our previous results show that their accuracies are 0.941 and 0.99 on the whole data, and 0.862 and 0.966 on the small class. Hence, the bagging-SMOTE and the non-framework models perform best on both the whole data and the small class samples. However, previous results on other datasets show that the bagging-SMOTE model always gets the highest accuracy in rare sample prediction, which can be attributed to its data distribution adjustment step during model training.

The result presubtypeclassifierres also contains a slot named “mod”, which is the final model trained from the whole dataset, and it can be transferred to another function *pairedensemblepredict*, which will use the model to predict other external samples.

In addition to the functions above, *CWGCNA* also has other functions to perform network-based gene functional enrichment (*diffwgcna* and *topoenrich*), differential feature identification (*difffeatures*), etc., and more details can be found in our package document and the paper of this package.

## References

1. Zhang B, Horvath S. A General Framework for Weighted Gene Co-Expression Network Analysis, Statistical Applications in Genetics and Molecular Biology 2005;4.
2. Dougan M, Dranoff G, Dougan SK. GM-CSF, IL-3, and IL-5 Family of Cytokines: Regulators of Inflammation, Immunity 2019;50:796-811.
3. Chang SH, Dong C. IL-17F: Regulation, signaling and function in inflammation, Cytokine 2009;46:7-11.
4. Bernucci L, Henriquez M, Diaz P et al. Diverse Calcium Channel Types are Present in the Human Placental Syncytiotrophoblast Basal Membrane, Placenta 2006;27:1082-1095.
5. Zhao Y, Pasanen M, Rysa J. Placental ion channels: potential target of chemical exposure, Biology of Reproduction 2022.
